# Supplementary material for: Drosophila females receive male substrate-borne signals through specific leg neurons during courtship
Source: Curr Biol. 2021 Sep 13;31(17):3894–3904.e5. doi: 10.1016/j.cub.2021.06.002 (PMC8445324; doi:10.1016/j.cub.2021.06.002)
Supplement: Document S1. Figures S1–S3 and Tables S1 and S2 [file mmc1.pdf]

Current Biology, Volume *31*

Supplemental Information

Drosophila females receive male substrate-borne signals through specific leg neurons during courtship

Eleanor G.Z. McKelvey, James P. Gyles, Kyle Michie, Violeta Barquín Pancorbo, Louisa Sober, Laura E. Kruszewski, Alice Chan, and Caroline C.G. Fabre

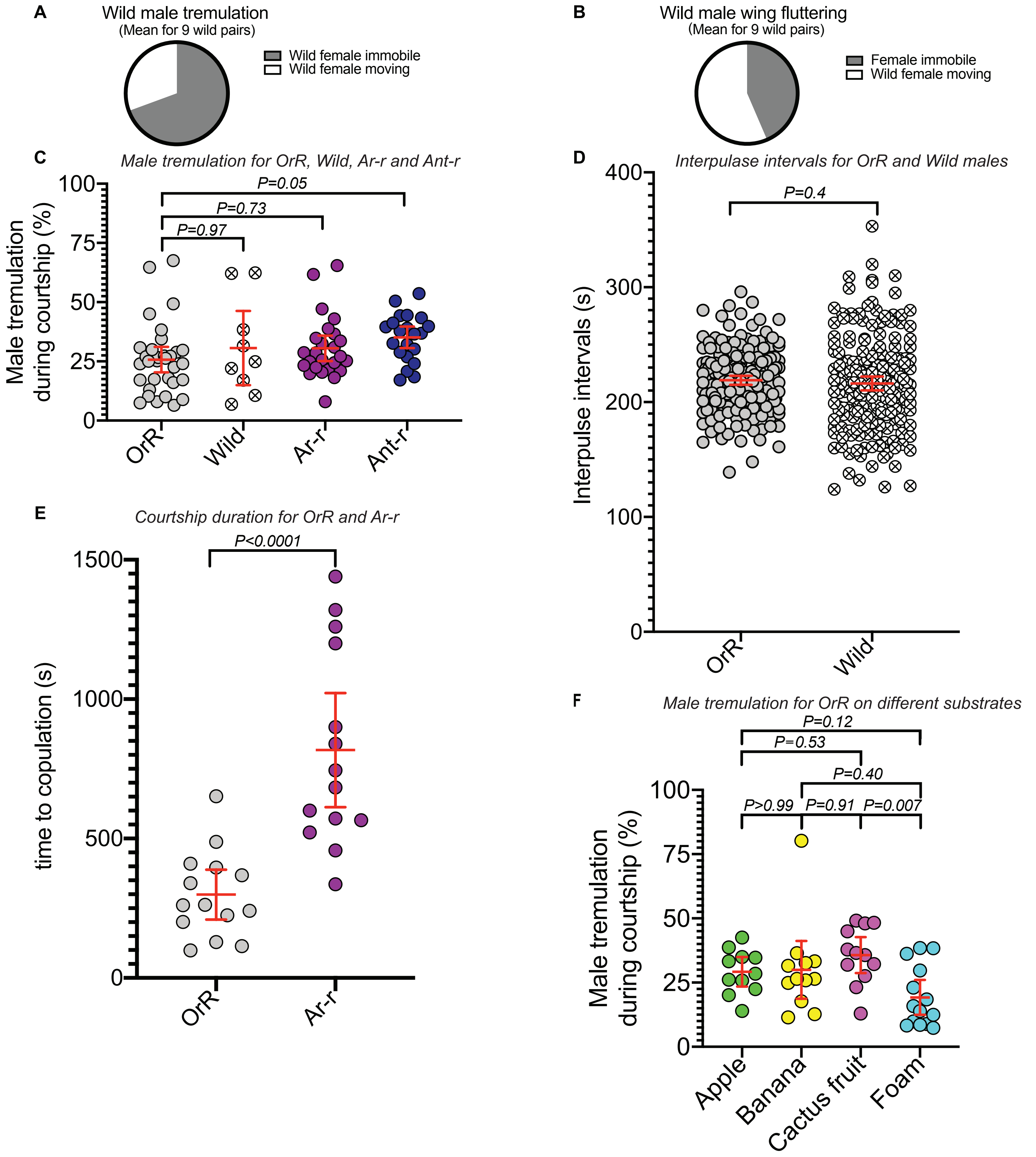

Figure S1. Details of the courtship of wild, OregonR, Ar-r and Ant-r *Drosophila* pairs, related to Figure 1. (A) and (B) compares quantification of the wild female movement with respect to whether the wild male is fluttering his wing or tremulating his abdomen during courtship (wild flies were collected in Italy, see STAR methods). (A) The pie chart shows the percentage of time where wild females were immobile or moving during wild male tremulations. Wild females spent on average 70 ± 7 % of the time being immobile during wild male tremulations. This is significantly higher than the time they spent immobile when the male was not tremulating (46 ± 7 %; see Figure 1C; P=0.03). (B) The pie chart shows the percentage of time where wild females were immobile or moving during wild male wing fluttering (producing the ‘love song’). Wild females spent around half of the time being immobile (56 ± 8 %) and half of the time moving during wild male wing fluttering. Wild male wing fluttering did not correlate strongly with female immobility (P=0.46), but wild male tremulations did, confirming findings with laboratory stocks [S1,S2]. (C) shows the total percentage of time where males were tremulating during courtship. This percentage in wild flies (n=9) and Ar-r pairs (n=25) is similar to that of intact OrR pairs (n=32); it is slightly higher in Ant-r pairs (n=22). (D) Interpulse intervals (in seconds) of the substrate-borne vibrations generated by tremulations of laboratory-stock OrR (n=188 pulses recorded) and of wild males (n=188 pulses recorded) recorded on the artificial foil during courtship with OrR females and wild females, respectively. Scatter plots are shown for 3 individu-als for each type. There were no significant differences in the mean interpulse interval between both. (E) shows the duration of court-ship preceding copulation for OrR pairs and for Ar-r pairs. It is significantly increased for Ar-r pairs presumably because *aristae-removed* females cannot receive the species-specific love song that promotes copulation. (F) shows the total percentage of time where OrR males were tremulating during courtship on natural and foam substrates for the same pairs as in Figure 1D-F. This percentage is similar on most substrates, but it differs significantly between cactus fruit and foam.

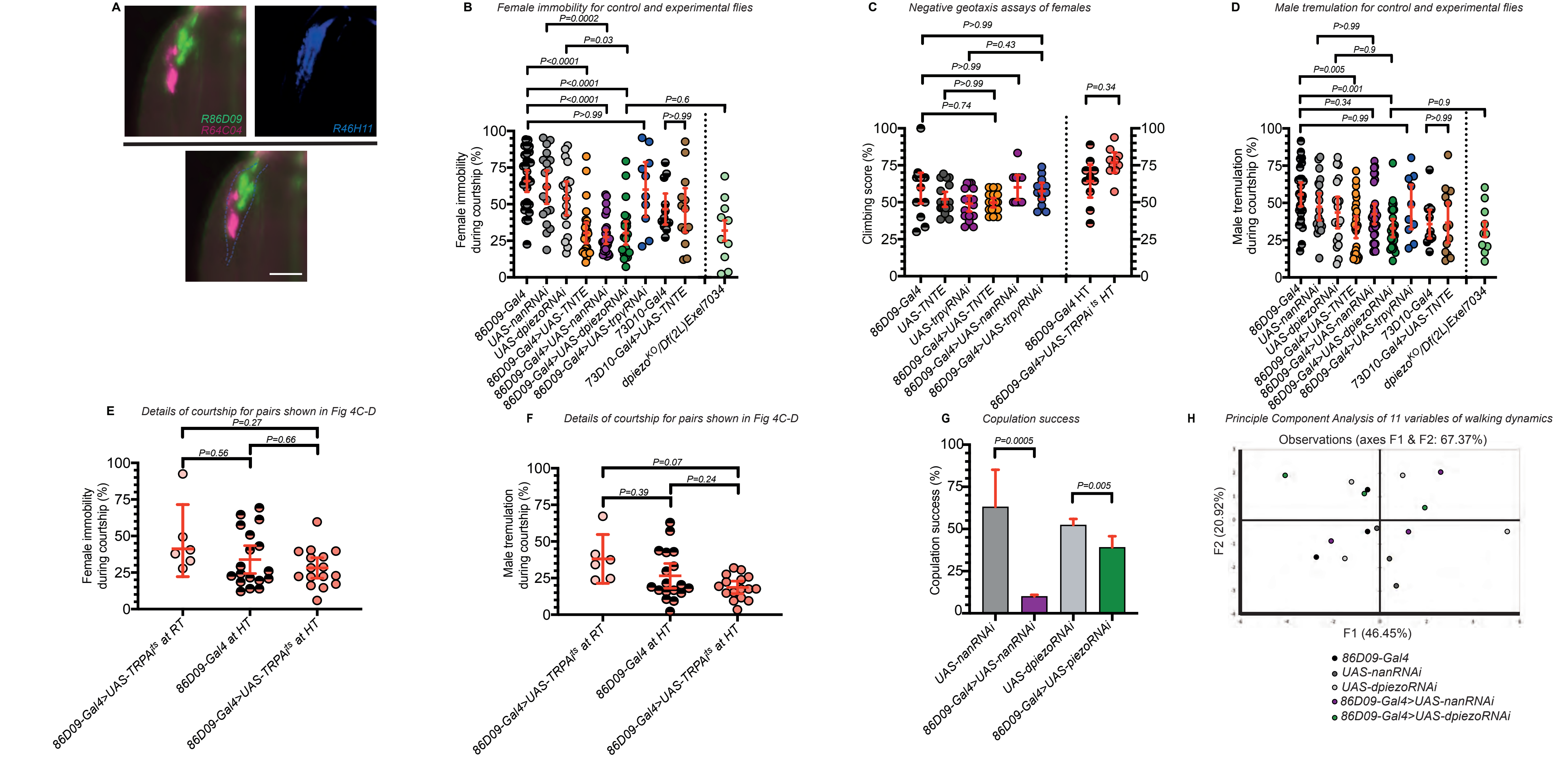

Figure S2. Details of expression, courtship and locomotor assays for experimental and control flies, related to Figure 4. (A) shows side view of three overlaid female legs (microscope z-stack projections); (left) shows overlaid expression of GFP under the control of either *R64C04-Gal4* (magenta; this line drives expression in around 30 club neurons[S3]) or *86D09-Gal4* (green); (right) shows expression of GFP under the control of *R46H11-Gal4* (blue; this line drives expression in a large portion of the club neurons[S4]); (bottom) shows overlay of the expression patterns for the three Gal4 lines (*R46H11-Gal4* expression is indicated using blue dashed lines); scale bar, 40µm (B) shows quantification of female immobility during courtship for pairs including control females carrying either *86D09-Gal4* or *73D10-Gal4*, or one of the UAS-RNAi lines, or a combination of both a Gal4 and a UAS, and pairs including a *dpiezo*^{KO}*/Df(2L)Exel7034* female. Ethograms were constructed from the same pairs shown in Figure 4A-B. Note that females carrying Gal4 and UAS construct show levels of immobility that differ from OrR females during courtship. We have no explanation for this phenomenon, which we have observed in our experimental set-up with all engineered lines carrying Gal4/UAS that we tested. *86D09-Gal4>UAS-TNTE*, *86D09-Gal4>UAS-nanRNAi*, *86D09-Gal4>UAS-dpiezoRNAi* all show significantly lower mean female immobility in comparison to their associated controls. *dpiezo*^{KO}*/Df(2L)Exel7034* females behaved similarly to *86D09-Gal4>UAS-dpiezoRNAi* females. *86D09-Gal4>UAS-trpγRNAi* females have similar immobility to their associated control. The mean value obtained for the immobility of *73D10-Gal4>UAS-TNTE* females is unchanged compared with *73D10-Gal4* control females. (C) Negative geotaxis climbing assays of females carrying a Gal4 or a UAS alone or in combination, using a tap down assay. Climbing scores were obtained from analysis of 14, 18, 17, 19, 19, 13, 10 and 10 climbing assays (in the order illustrated on the graph). Assays displayed to the right of the dashed line were performed at high temperature. No climbing defect could be observed in any of the females tested. Note that adult knockouts for the gene *dpiezo* have previously been shown to have a normal locomotion in climbing assays[S5]. (D) shows the total percentage of time where males were tremulating during courtship for the same pairs as in (A) and Figure 4A-B. Males tremulate less when paired with females *86D09-Gal4>UAS-TNTE* and *86D09-Gal4>UAS-dpiezoRNAi*. In all other pairs males displayed similar level of tremulations to associated controls. (E) shows quantification of experimental and control female immobility during courtship in the same conditions and for the same pairs as in Figure 4C-D. Female immobility during courtship is similar in all 3 types of pairs. (F) shows the total percentage of time where males were tremulating during courtship for the same pairs as in (D) and Figure 4C-D. This percentage is similar in all 3 types of pairs. Note that males tremulate at a different frequency than that observed for males in OrR pairs, which we suppose is a response to the level of immobility and receptivity of females carrying the constructs (see B), courtship being an interactive behaviour between mates. (G) The percentage of copulation success is shown for pairs including control females carrying either *UAS-nanRNAi* (n=19) or *UAS-dpiezoRNAi* (n=21), and experimental females carrying the combination of *86D09-Gal4* with one of these *UAS-RNAi* (n=30 and n=23, respectively). This percentage is significantly lower in pairs including the experimental flies, suggesting that interfering with these ion channels in *86D09-Gal4*-neurons also altered mating. (H) Principle Component Analysis (PCA) of 11 variables of walking dynamics measured in a walking assay and comparing across females carrying either *86D09-Gal4*, *UAS-nanRNAi, UAS-dpiezoRNAi*, *86D09-Gal4>UAS-dpiezoRNAi* or *86D09-Gal4>UAS-nanRNAi*. 3/4 flies were tracked for each genotype and each dot represents the data point for a specific fly. The PCA shows 67.37% of the total variability in the data set. There is no obvious clustering of the data points, which suggests that locomotion does not vary depending on the constructs present in the female. Differences display solely variations between flies, independently of their genotype.

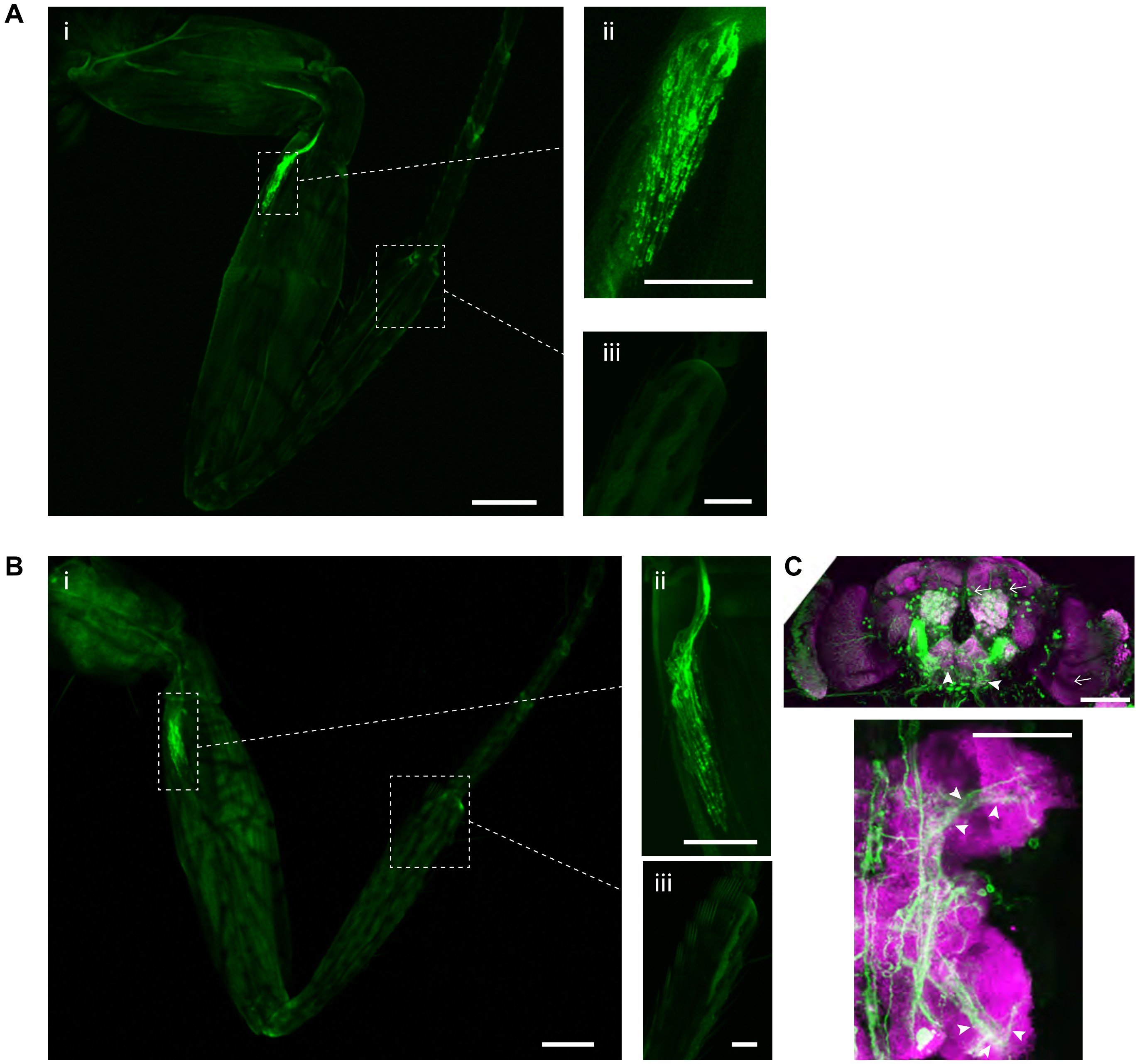

Figure S3. Pattern of expression of the Gal4 lines *Nan-Gal4* and *dPiezo-Gal4* in the leg and in the central nervous system, related to Figure 4. (A) A confocal image of the front leg of a female carrying the construct *Nan-Gal4>UASmCD8GFP.* (i) shows expression (bright green) in the femoral chordotonal organ and no expression in the tibial chordotonal organ; the axons of the femoral chordotonal neurons bundle to project upwards towards the trochanter and the central nervous system. Scale bar indicates 100 µm; (ii) shows expression in hundreds of cell bodies and processes of the femoral chordotonal organ (bright green). Scale bar indicates 40 µm; (iii) shows no expression where the tibial chordotonal organ is localised. Scale bar indicates 40 µm. Light green is autofluorescence from the cuticle. Note that, in[S6,], the adult brain of *Nan-Gal4>UAS-mCD8GFP* does not display GFP in the optic lobes nor in the ellipsoid body or ring neurons where *86D09-Gal4* also displayed some expression. (B) A confocal image of the front leg of a female carrying the construct *dPiezo-Gal4>UAS-mCD8GFP*. (i) shows expression (bright green) in the femoral chordotonal organ and no expression in the tibial chordotonal organ. Scale bar indicates 100 µm; (ii) shows expression in hundreds of cell bodies and processes of the femoral chordotonal organ (bright green). The axons of the neurons bundle to project upwards towards the trochanter and the central nervous system. Scale bar indicates 40 µm; (iii) shows no expression where the tibial chordotonal organ is localised. Scale bar indicates 40 µm. Light green is autofluorescence from the cuticle. (C) A confocal image showing axon terminals (green) of *dPiezo-Gal4>UAS-mCD8GFP*-expressing neurons in a female fly brain (top) and ventral nerve cord (VNC, bottom) labelled by the neuropil marker NC82 (magenta). (top) In the brain small processes targeting the anterior side of the brain can be distinguished in the gnathal ganglion (arrowheads). They resemble descriptions of some femoral chordotonal neuron projections that target directly this region of the brain[S8]. GFP is not visible in the optic lobes nor in the ellipsoid body or ring neurons where *86D09-Gal4* also displayed some expression (arrows; and see Extended Data in[S8]); (bottom) In the ventral nerve cord (only the first two hemi-neuropils are displayed), projections display the characteristic club shape of femoral chordotonal neuron projections (arrowheads); they are localised in the most dorsal layer in the middle of the neuropil of the thoracic ganglia that is also characteristic of the fCHO club neurons of the femoral chordotonal organ[S3,S9]. Scale bars indicate 100 µm. Note, *dPiezo-Gal4*[S5] may not perfectly represent *dpiezo* expression as it does not include the complete endogeneous promoter but includes only 1000bp of upstream promoter sequence[S5]; its expression pattern in the adult brain appears, in general, similar to the expression pattern of a recently made *Trojan-dpiezo-Gal4* line (http://flypush.imgen.bcm.tmc.edu/pscreen/rmce/rmce.php?entry=RM00971), suggesting that the expression pattern of *dpiezo-Gal4*[S5] is broadly reflective of endogenous *dpiezo* expression. Thus, while we cannot completely rule out that endogenous *dpiezo* could be expressed within brain neurons targeted by *86D09-Gal4*, the most parsimonious explanation for our results presented in Figure 4 is that it is the depletion of *dpiezo* within the *86D09-Gal4* fCHO neurons, rather than its depletion within the *86D09-Gal4*-expressing brain neurons, that leads to the observed phenotype.

**Comparison of female immobility for OrR, Ar-r and Ant-r pairs depending on whether the male is tremulating or not**					
Dunnett's T3 multiple comparisons test	Mean Diff.	95.00% CI of diff.	Significant?	Summary	Adjusted P Value
IT OrR vs. INT OrR	26.96	11.36 to 42.56	Yes	****	<0.0001
IT Ar-r vs. INT Ar-r	15.46	-0.4716 to 31.39	Yes	*	0.0338
IT Ant-r vs. INT Ant-r	41.27	25.23 to 57.31	Yes	****	<0.0001
**Comparison of female immobility on different substrates depending on whether the male is tremulating or not**					
Dunnett's T3 multiple comparisons test	Mean Diff.	95.00% CI of diff.	Significant?	Summary	Adjusted P Value
IT Apple vs. INT Apple	14.25	-2.991 to 31.48	No	ns	0.1056
IT Banana vs. INT Banana	23.8	-1.102 to 48.69	Yes	*	0.0367
IT Cactus fruit vs. INT Cactus fruit	18.82	-0.2839 to 37.92	Yes	*	0.0285
IT Foam vs. INT Foam	-44.66	-74.23 to -15.09	Yes	***	0.0003
**Comparison of female immobility in control and experimental pairs depending on whether the male is tremulating or not**					
Dunnett's T3 multiple comparisons test	Mean Diff.	95.00% CI of diff.	Significant?	Summary	Adjusted P Value
IT 86D09-Gal4 vs. *INT 86D09-Gal4*	67.89	54.67 to 81.12	Yes	****	<0.0001
IT UAS-TNTE vs. *INT UAS-TNTE*	43.77	28.70 to 58.85	Yes	****	<0.0001
IT UAS-nanRNAi vs. *INT UAS-nanRNAi*	56.32	43.06 to 69.59	Yes	****	<0.0001
IT UAS-piezoRNAi vs. *INT UAS-piezoRNAi*	48.35	28.33 to 68.37	Yes	****	<0.0001
IT 86D09-Gal4>UAS-TNTE vs. *INT 86D09-Gal4>UAS-TNTE*	21.06	-0.4707 to 42.58	Yes	*	0.03
IT 86D09-Gal4>UAS-NanRNAi vs. *INT 86D09-Gal4>UAS-nanRNAi*	15.26	-0.6176 to 31.15	Yes	*	0.04
IT 86D09-Gal4>UAS-piezoRNAi vs. *INT 86D09-Gal4>UAS-piezoRNAi*	28.38	5.534 to 51.22	Yes	**	0.001
IT 86D09-Gal4>UAS-trpγRNAi vs. *INT 86D09-Gal4>UAS-trpγRNAi*	64.24	49.30 to 79.18	Yes	****	<0.0001
IT 73D10-Gal4 vs. *INT 73D10-Gal4*	50.09	29.03 to 71.16	Yes	****	<0.0001
IT 73D10-Gal4>UAS-TNTE vs. *INT 73D10-Gal4>UAS-TNTE*	62.32	40.71 to 83.92	Yes	****	<0.0001
IT *dpiezo*^{KO}*/Df(2L)Exel7034* vs. INT*dpiezo*^{KO}*/Df(2L)Exel7034*	-3.588	-51.61 to 44.43	No	ns	>0.9999

Table S1. Comparison of female immobility during courtship depending on whether the male is tremulating or not, related to Figures 1 and 4. In the first column, IT stands for "Immobility during male Tremulation", and INT for "Immobility when the male is Not Tremulating". The **top panel** shows statistical data for OrR, Ar-r and Ant-r pairs. A Dunnett's T3 multiple comparisons test was applied to values displayed in figure 1B and 1C, to verify if female immobility varied significantly whether the male tremulated or not. P values are presented in the table. They show that female immobility is significantly different in the OrR, Ar-r and Ant-r pairs depending on whether the male tremulates or not. In the 3 types of pairs the female is significantly more immobile when the male tremulates. The **middle panel** shows statistical comparison of female immobility on different substrates depending whether the male is tremulating or not. A Dunnett's T3 multiple comparisons test was applied to values displayed in figure 1E and 1F, to verify if female immobility on one substrate varied significantly whether the male tremulated or not. P values show that female immobility during tremulation is significantly different on the banana, cactus fruit and foam compared to immobility when the male is not tremulating. The **bottom panel** shows statistical data using a Dunnett's T3 multiple comparisons test applied to values displayed in figure 4A and 4B, to verify if control and experimental female immobility varied significantly whether the male tremulated or not. P values show that female immobility is significantly different depending on whether the male tremulates or not (female is significantly more immobile when the male tremulates), but less so when the females are carrying the constructs *86D09-Gal4>UAS-TNTE*, *86D09-Gal4>UAS-nanRNAi* or *86D09-Gal4>UAS-dpiezoRNAi.* This suggests that the movement of these three types of females is tending towards being independent of the tremulations. The immobility of females *dpiezo*^{KO}*/Df(2L)Exel7034* is similar, whether the male is tremulating or not.

Substrate	Fly falling on the substrate from the opposite side of the chamber	Fly walking on the substrate	Substrate-borne vibrations generated by tremulations	Wing fluttering substrate-borne components	Number of flies recorded
Stone	+	-	-	-	5
Wood slice	+	-	-	-	3
Banana	+	+	+	+	10
Apple	+	+	+	+	10
Prickly Pear Cactus fruit	+	+	+	+	15
Foil	+	+	+	+	20
Isolating Foam	-	-	-	-	3

Table S2. Types of signals picked up by the laser doppler vibrometer during *D. melanogaster* OrR courtship on different substrates, related to Figure 2. In all cases the beam of the laser was targeted onto the substrate around the middle of the courtship area of similar size and substrate thickness (see STAR methods). “Wing fluttering substrate-borne components” refers to the signals generated by wing fluttering in the substrate recently described by[S10]. The + indicates that signal was picked up by the laser, - indicates that no signal was visible on the recordings.

Supplemental References

S1. Fabre, C.C.G., Hedwig, B., Conduit, G., Lawrence, P.A., Goodwin, S.F., and Casal, J. (2012). Substrate-borne vibratory communication during courtship in *Drosophila* melanogaster. Curr. Biol. *22*, 2180–2185.

S2. Medina, I., Casal, J., and Fabre, C.C.G. (2015). Do circadian genes and ambient temperature affect substrate-borne signalling during *Drosophila* courtship? Biol. Open *4*, 1549–1557.

S3. Mamiya, A., Gurung, P., and Tuthill, J.C. (2018). Neural coding of leg proprioception in *Drosophila*. Neuron *100*, 636–650.

S4. Jenett, A., Rubin, G.M., Ngo, T.T.B., Shepherd, D., Murphy, C., Dionne, H., Pfeiffer, B.D., Cavallaro, A., Hall, D., Jeter, J., et al. (2012). A Gal4-driver line resource for *Drosophila* neurobiology. Cell Rep. *2*, 991–1001.

S5. Kim, S.E., Coste, B., Chadha, A., Cook, B., and Patapoutian, A. (2012). The role of *Drosophila* Piezo in mechanical nociception. Nature *483*, 209–212.

S6. Jourjine, N., Mullaney, B.C., Mann, K., and Scott, K. (2016). Coupled sensing of hunger and thirst signals balances sugar and water consumption. Cell *166*, 855–866.

S7. Woo, S.H., Ranade, S., Weyer, A.D., Dubin, A.E., Baba, Y., Qiu, Z., Petrus, M., Miyamoto, T., Reddy, K., Lumpkin, E.A., et al. (2014). Piezo2 is required for Merkel-cell mechanotransduction. Nature *509*, 622–626.

S8. Ramdya, P., Lichocki, P., Cruchet, S., Frisch, L., Tse, W., Floreano, D., and Benton, R. (2015). Mechanosensory interactions drive collective behaviour in *Drosophila*. Nature *450*, 294-298.

S9. Tsubouchi, A., Yano, T., Yokoyama, T.K., Murtin, C., Otsuna, H., and Ito, K. (2017). Topological and modality-specific representation of somatosensory information in the fly brain. Science (80-.). *358*, 615–623.

S10. Mazzoni, V., Anfora, G., and Virant-Doberlet, M. (2013). Substrate vibrations during courtship in three *Drosophila* species. PLoS One *8(11)*.
