# Supplementary material for: Drosophila females receive male substrate-borne signals through specific leg neurons during courtship
Source: Curr Biol. 2021 Sep 13;31(17):3894–3904.e5. doi: 10.1016/j.cub.2021.06.002 (PMC8445324; doi:10.1016/j.cub.2021.06.002)
Supplement: Document S2. Article plus supplemental information [file mmc5.pdf]

Report

Current Biology

Drosophila females receive male substrate-borne signals through specific leg neurons during courtship

Graphical abstract

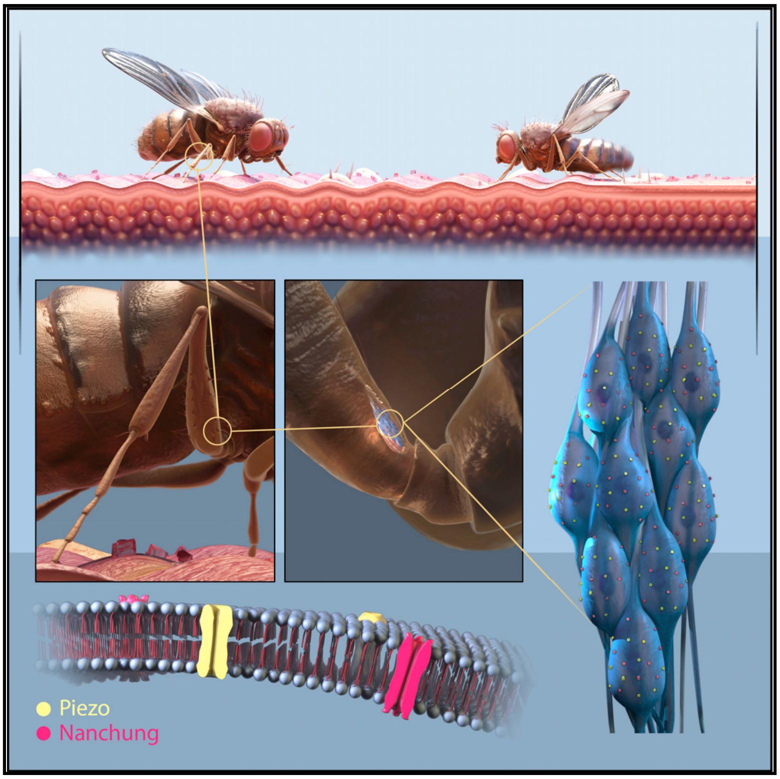

Highlights

- Courting male abdominal tremulations signal via substrate-borne, not air-borne, vibrations
- Substrate-borne vibrations propagate through natural food substrates
- Neurons of the leg fCHO mediate female immobility response to these vibrations
- Mechanically gated ion channels Nan and Piezo mediate response in these neurons

Authors

Eleanor G.Z. McKelvey,
James P. Gyles, Kyle Michie, ...,
Laura E. Kruszewski, Alice Chan,
Caroline C.G. Fabre

Correspondence

c.c.g.fabre.03@cantab.net

In brief

McKelvey et al. find that courting male *D. melanogaster* abdominal tremulations signal via substrate-borne, not air-borne, vibrations. These vibrations propagate through natural food substrates, promoting female immobility. Neurons and their mechanically gated ion channels within the leg fCHO mediate female response to the vibrations.

McKelvey et al., 2021, Current Biology *31*, 3894–3904
September 13, 2021 © 2021 The Author(s). Published by Elsevier Inc.
https://doi.org/10.1016/j.cub.2021.06.002

CellPress

CellPress
OPEN ACCESS

Current Biology

Report

Drosophila females receive male substrate-borne signals through specific leg neurons during courtship

Eleanor G.Z. McKelvey,[1] James P. Gyles,[1] Kyle Michie,[1] Violeta Barquín Pancorbo,[1] Louisa Sober,[1] Laura E. Kruszewski,[1] Alice Chan,[1] and Caroline C.G. Fabre[1,2,3,*]
[1]Department of Zoology, University of Cambridge, Downing Street, Cambridge CB2 3EJ, UK
[2]Twitter: @CarolineFabre9
[3]Lead contact
*Correspondence: c.c.g.fabre.03@cantab.net
https://doi.org/10.1016/j.cub.2021.06.002

SUMMARY

Substrate-borne vibratory signals are thought to be one of the most ancient and taxonomically widespread communication signals among animal species, including *Drosophila* flies.[1–9] During courtship, the male *Drosophila* abdomen tremulates (as defined in Busnel et al.[10]) to generate vibrations in the courting substrate.[8,9] These vibrations coincide with nearby females becoming immobile, a behavior that facilitates mounting and copulation.[8,11–13] It was unknown how the *Drosophila* female detects these substrate-borne vibratory signals. Here, we confirm that the immobility response of the female to the tremulations is not dependent on any air-borne cue. We show that substrate-borne communication is used by wild *Drosophila* and that the vibrations propagate through those natural substrates (e.g., fruits) where flies feed and court. We examine transmission of the signals through a variety of substrates and describe how each of these substrates modifies the vibratory signal during propagation and affects the female response. Moreover, we identify the main sensory structures and neurons that receive the vibrations in the female legs, as well as the mechanically gated ion channels Nanchung and Piezo (but not Trpγ) that mediate sensitivity to the vibrations. Together, our results show that *Drosophila* flies, like many other arthropods, use substrate-borne communication as a natural means of communication, strengthening the idea that this mode of signal transfer is heavily used and reliable in the wild.[3,4,7] Our findings also reveal the cellular and molecular mechanisms underlying the vibration-sensing modality necessary for this communication.

RESULTS AND DISCUSSION

Drosophila melanogaster wild flies exhibit substrate-borne communication signals similar to laboratory fly stocks

Substrate-borne vibratory signals during courtship have been reported in *D. melanogaster* laboratory stocks,[8,9,11] but not in wild *D. melanogaster*. Single wild males courted single wild females vigorously, including wing fluttering and abdominal tremulations (Video S1), and the wild females responded similarly to Oregon-R laboratory stocks (Figures 1A–1C and S1A–S1C).[8,9,11] The durations of the interpulse intervals (IPIs) are often used by animals for signal recognition;[1,4,7,14–19] we used laser vibrometry to measure and compare the IPI of the substrate-borne vibrations produced by wild and laboratory male's abdominal tremulations and found them to be similar to one another (Figure S1D). Therefore, all our later experiments were performed on laboratory Oregon-R flies.

The signals produced by male abdominal tremulations, and received by the female to promote her immobility, are not air borne

During courtship, a number of cues convey information about the pairs' identity and fitness,[20–25] particularly the reproductive, nutritional, and receptive status of the female.[21,23] Chemical, visual, and air-borne signals modify the behavior of the male and the responsiveness of the female.[21–30] *D. melanogaster* courtship relies on a near-field air-borne signal, the "love song," which is produced by the male's wing fluttering.[21–28] We surgically removed the whole antennae or only the aristae (essential for air-borne sound reception)[26] from females, paired them with normal males, studied their courtship, and compared it with the courtship of intact Oregon-R couples (Figures 1A–1C, S1C, and S1E). In all three treatments, female immobility strongly coincided with bouts of male tremulation (Figure 1B), and female immobility was low when the male did not tremulate (Figures 1B and 1C; Table S1). These data are consistent with the hypotheses that female immobility is not regulated by air-borne signals and that females do not detect males' tremulations via air-borne signals.

Drosophila vibrations' propagation varies through natural substrates, but the females' responses are similar

The fidelity of transmission of the substrate-borne signals and the response to those signals may depend heavily on the physical

 © 2021 The Author(s). Published by Elsevier Inc.
This is an open access article under the CC BY license (http://creativecommons.org/licenses/by/4.0/).

CellPress
OPEN ACCESS

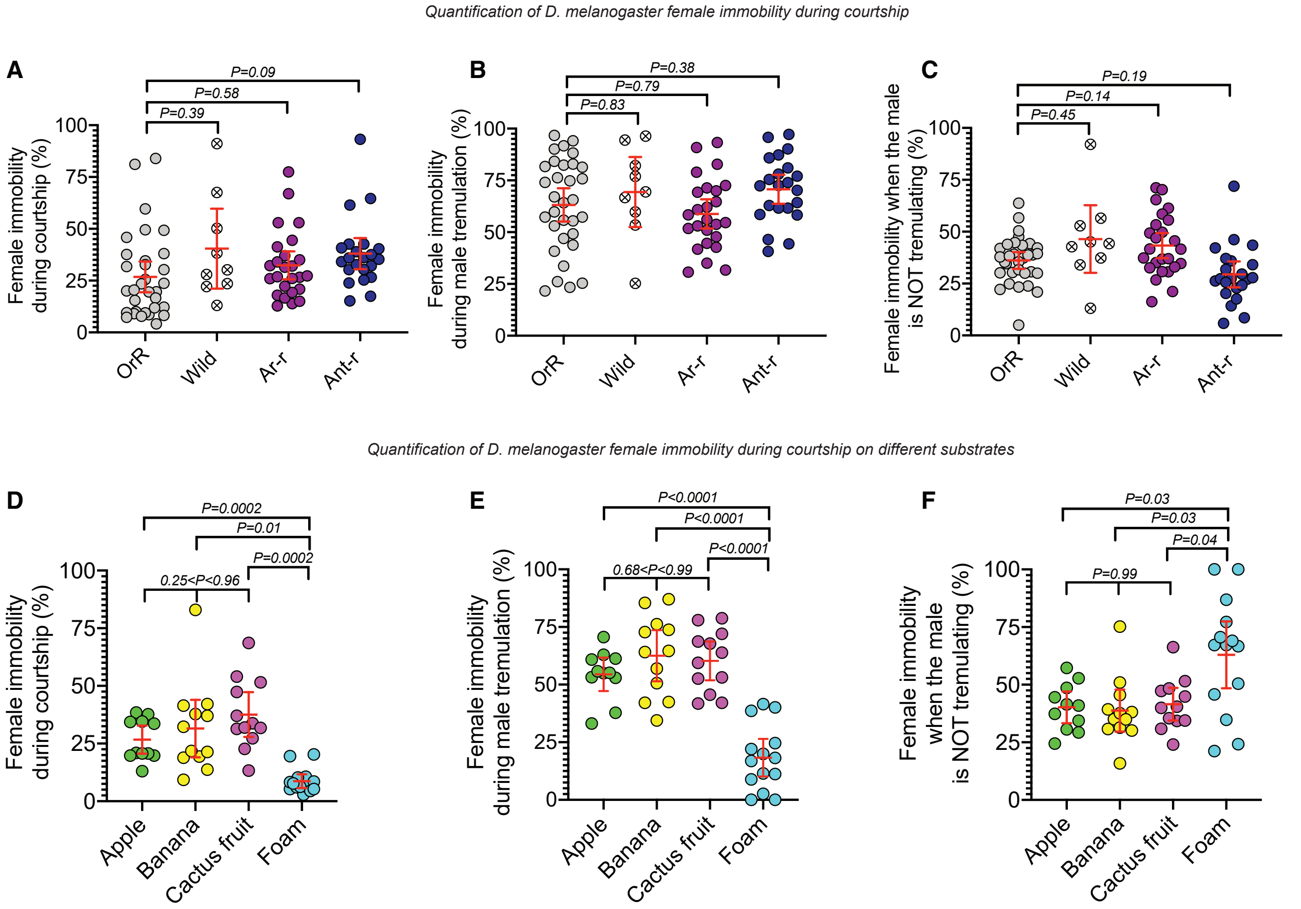

Figure 1. Quantification of *D. melanogaster* female immobility during courtship

(A–C) Data for intact OregonR (OrR) pairs, wild flies, and *aristae-removed* or *antennae-removed* OrR females paired with intact OrR males (Ar-r pairs and Ant-r pairs, respectively) filmed in plastic chambers. Ethograms constructed from analysis of video clips of 32, 9, 25, and 22 pairs, respectively.

(A) The total percentage of time females were immobile during courtship is similar for all pairs.

(B) The percentage of time where females were immobile while the male abdomen was tremulating is similar for all pairs. Note that, in all cases, the male was tremulating for a similar duration during courtship (Figure S1C).

(C) The percentage of time where females were immobile while the male abdomen was not tremulating is similar in all pairs.

(D–F) Data for OrR pairs on different substrates, including apple, banana, cactus fruit, or foam. Ethograms are constructed from analysis of 11, 12, 12, and 14 pairs, respectively.

(D) The total percentage of time females were immobile during courtship is significantly lower on foam (9% ± 1%) compared to the other substrates (27% ± 3%, 31% ± 6%, and 38% ± 4%, respectively), where it is similar.

(E) The percentage of time where females were immobile while the male tremulated was similar on apple (54% ± 3%), banana (62% ± 5%), and cactus (60% ± 4%) but significantly lower on foam (18% ± 4%).

(F) The percentage of time where females were stationary while the male was not tremulating his abdomen is similar on the natural substrates (40% ± 3%, 39% ± 4%, and 41% ± 3%, respectively) but significantly higher on foam (63% ± 7%). There is no significant difference between OrR female immobility in plastic chambers and OrR female immobility on apple, banana, and cactus fruit ($p > 0.99$, $p = 0.86$, and $p = 0.19$, respectively), suggesting that the presence of an edible substrate does not modify female's immobility and response to the vibrations during courtship.

See also Figure S1, Table S1, and Video S1.

properties of the courting substrates.[31–39] So far, laser vibrometry has been used to study *Drosophila* substrate-borne vibrations on reflective materials.[8,9,11,40] *Drosophila* typically meet and court on soggy and rotten fruits.[41–44] By recording vibrations on apple, banana, and prickly pear cactus fruits, we found that these natural substrates propagate fly vibrations (Table S2; Audio S1). Signal amplitude was highest on cactus fruits (Figure 2A; Audio S1). Our laser vibrometer did not record substrate vibrations on stone and wood, even though these substrates are used for signaling by spiders and termites,[35,45–48] nor on insulating foam material (Table S2).[39,49]

We next investigated the physical parameters of the vibrations. We analyzed the IPIs of male tremulations transmitted through the various substrates. The IPI of the vibrations generated on cactus fruits (258 ± 5 ms) were significantly different from those on banana (204 ± 6 ms) and apple (207 ± 9 ms; Figure 2B). These results suggest that properties of the substrates, such as viscosity, may affect the male's ability to raise his

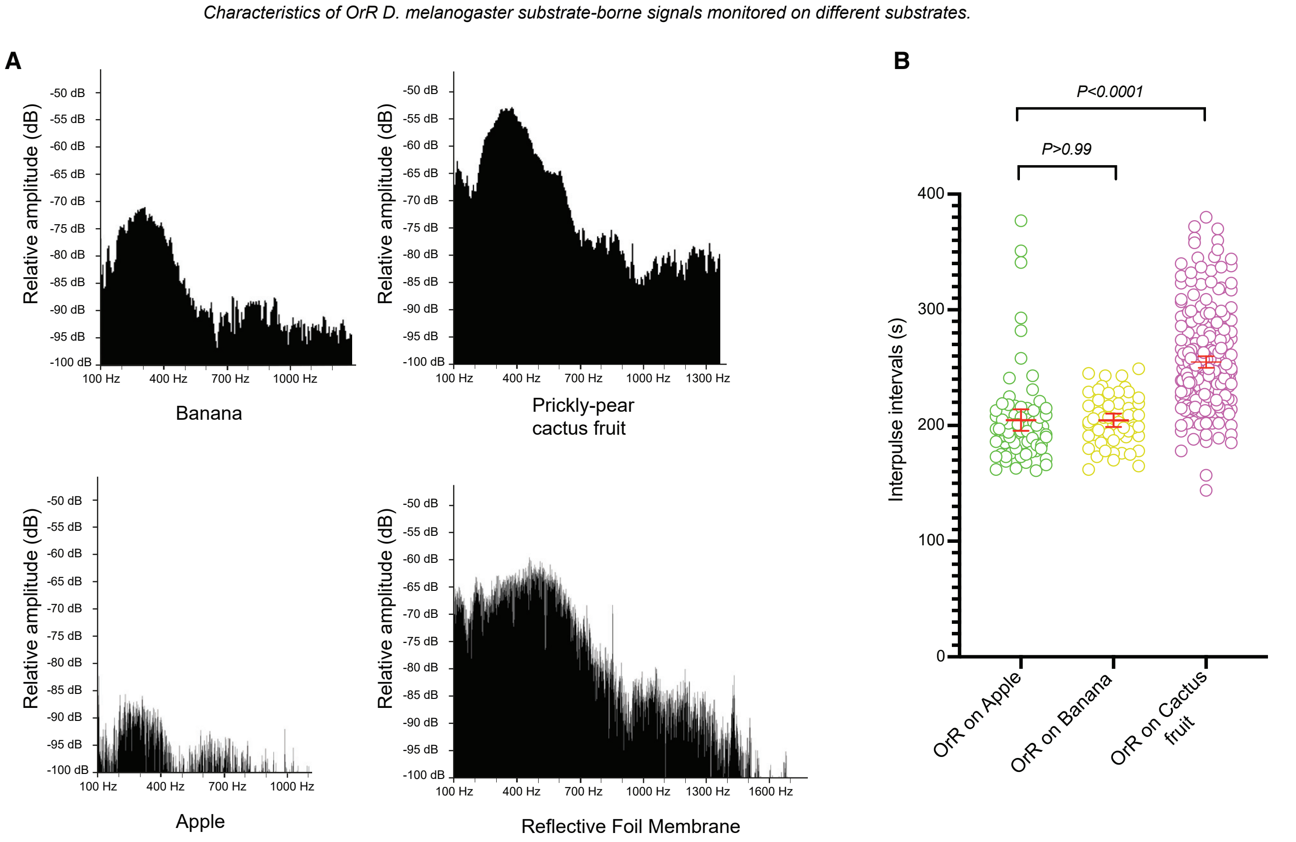

Figure 2. Characteristics of *D. melanogaster* substrate-borne vibrations monitored on different substrates during courtship

(A) Pattern of frequencies and amplitude of 1–3 vibratory pulses generated by tremulations of OrR males recorded on banana, apple, prickly-pear cactus fruit, or on the artificial foil membrane during courtship with OrR females.

(B) Interpulse intervals (IPIs) of the substrate-borne vibrations of OrR males on apple (n = 74 pulses recorded, 2 flies), banana (n = 55 pulses recorded, 2 flies), and cactus fruit (n = 251 pulses recorded, 4 flies). The mean IPIs recorded on apple and banana were similar, but they were significantly different from the mean IPI recorded on the cactus fruits.

See also Table S2.

abdomen to tremulate, and the properties of the cactus fruits may cause the males to tremulate in a unique fashion. The repetition rate was near 3.9 Hz (for cactus fruits) and around 4.9 Hz (for banana and apple); these values are similar to those measured when we modified other parameters in the environment, such as the ambient temperature.[11] Large differences in the pulse repetition rate of vibratory signals have previously been reported for different *Drosophila* species,[8,9,40] some of which share food substrates. For example, the prickly pear cactus is a natural breeding and courting ground for several species of *Drosophila*.[41,43,50–52] These large differences may have a role in intraspecific courtship communication to avoid interspecific breeding, as in other vibratory insects (see, for example, Hrabar et al.[53] and Miklas et al.[54]).

Substrate-borne signals are also characterized by the frequency spectra and the dominant frequencies of vibratory pulses, which may vary on different substrates.[3,32,37,55–57] Both of these measures were complex (Figure 2A): on the reflective foil membrane, the spectrum of a vibratory unit showed a broad peak of high amplitude at frequencies between 200 Hz and 800 Hz, with a peak around 500 Hz. Another broad peak of lower amplitude was visible at frequencies between 1,000 Hz and 1,300 Hz. On the cactus fruit, values showed high amplitude from 200 Hz to 600 Hz, with a peak at about 400 Hz. On banana, we observed a narrow frequency peak around 300 Hz, and the amplitude of the signal was much lower than that observed on cactus fruits. The signal obtained on apple was similar but of even lower amplitude. On apple and banana, frequencies above 1,000 Hz did not display another peak (Figure 2A). These results suggest that *Drosophila* natural courting substrates modify the spectral properties of the signals and act as filters, attenuating the high frequencies.[6,32,58–60] Herbaceous plants have similar properties, also acting as low pass filters.[33,61–63]

We studied the behavior of courting flies on these substrates. Females remained immobile for a similar percentage of time during courtship on all fruits but moved more on foam (Figure 1D). There was a strong correlation between male tremulation and female immobility on all the fruits, but not on foam (Figures 1E and 1F; Table S1). These results show that substrate-borne signals are effective through a variety of fruit substrates, regardless of the different frequency and IPI patterns (Figure 2), arguing that these signal variations fit within the sensitivity range of the receiving sensory structures. On foam, a striking impairment of female responsiveness (Figures 1D–1F) suggests a lack of

CellPress
OPEN ACCESS

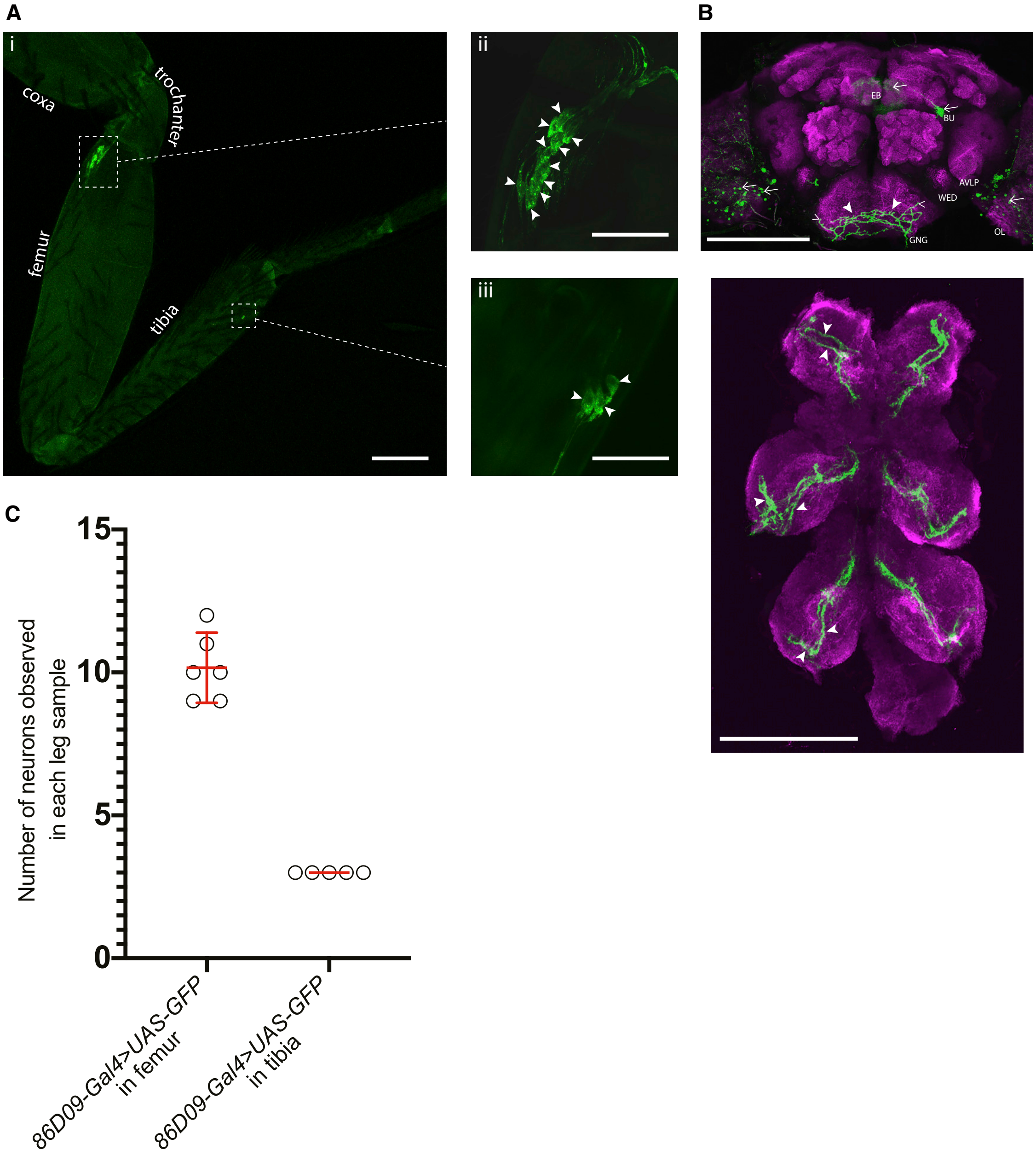

Figure 3. Pattern of expression of the *86D09-Gal4* line in the leg and in the central nervous system

(A) Confocal image of the front leg of a *86D09-Gal4>UAS-mCD8GFP* female; (i) expression (bright green) in the fCHO and the tCHO, scale bar, 100 μm; (ii) expression in 10 cell bodies of the fCHO (bright green; arrowheads) and in their associated neurites that bundle to project upward toward the trochanter and the central nervous system (CNS) and downward toward the cuticle,[74] scale bar, 40 μm; (iii) expression in the cell bodies (arrowheads) of the 3 tCHO neurons, as well as in their axons bundling to project toward the leg nerve and the CNS. Scale bar, 40 μm. Light green is autofluorescence from the cuticle.

(B) Confocal image showing axon terminals (green) of *86D09-Gal4>UAS-mCD8GFP* neurons in a female brain (top) and ventral nerve cord (VNC) (bottom) labeled by the neuropil marker NC82 (magenta). Top: the processes targeting the anterior side of the brain in the gnathal ganglion (GNG) (arrowheads) resemble descriptions that a few fCHO neurons target directly this region of the brain and pursue anterior-dorsally along the lateral side of the gnathal ganglion (thin arrowheads). These processes are not seen continuing deeper in the brain toward the wedge neuropil (WED) and the ventral-most part of the anterior ventrolateral protocerebrum (AVLP),[82] probably due to the fact that only a small subset of neurons is stained in comparison to Tsubouchi et al.;[82] GFP is also seen in neuron cell bodies in the optic lobes (OLs) (arrows; they resemble retinal neurons), in a pair of bilateral neurons of the posterior brain in the bulb region (BU) that resemble $PB_{G1\text{-}8}$.b-EBw.s-D/VGA.b ring neurons that respond to visual stimuli,[90–92] and weakly in the ellipsoid body (EB) where ring neurons project (arrow) and which is a central brain region for visual processing.[90] Female's vision is not necessary for her immobility response to the tremulations,[8] but we cannot fully exclude that these regions known for visual processing may also be involved in vibratory signal processing. Bottom: two thin sets of axon projection bundles (arrowheads in

(legend continued on next page)

propagation of male substrate-borne signals.[39] This impairment could possibly result from the taste and odor of the foam, which could alter the female's multisensory integration of male cues through her gustatory and olfactory receptor neurons,[23] although we did not observe such a lack of responsiveness in plastic chambers where vibratory signals can propagate[8,39] nor with antennaeless females (this report). The total time the male spent tremulating was significantly higher on cactus than on foam (Figure S1F), suggesting that the male may increase tremulation when on a conducive substrate, perhaps in reaction to responses from the female.[64–66]

The signals produced by abdominal tremulations are received by females via specific leg chordotonal neurons

Next, we asked which organs and neurons might act as vibration receptors in flies. In other arthropods, chordotonal organs within the legs detect substrate-borne vibrations, particularly the subgenual organ, which is absent in *Drosophila*.[7,48,67–73] Flies do, however, possess a chordotonal organ in the femur (the fCHO) and also in the tibia (the tCHO). Their location and anatomy make them candidates for the reception of substrate-borne vibrations during *Drosophila* courtship;[74–76] indeed, the calcium responses of a subset of fCHO "club" neurons showed that these neurons respond to artificial vibratory stimuli in addition to bidirectional movements of the tibia (the latter relates to a role in proprioception and locomotion).[75] Also, fCHO club neurons project toward the thoracic ganglions, where central interneurons *10Bα* respond to vibrations and mediate female immobility.[77] To investigate whether neurons in the female legs respond to the vibrations, we reviewed Gal4 lines[78] that expressed within distinct subsets of the CHO neurons in the leg, and we tested them by driving the expression of a neuronal inhibitor (*UAS-TNTE*; during metamorphosis and adulthood)[79–81] in females, in courtship assays. One of these lines was *86D09-Gal4.* We used membrane-bound GFP in combination with *86D09-Gal4* to observe expression in the periphery (Figure 3A) and in the brain and ventral nerve cord (VNC) (Figure 3B). We identified ~10 neurons in the fCHO and 3 neurons in the tCHO (Figures 3A and 3C). *86D09-Gal4* neuron projections in the gnathal ganglion of the brain resemble those described for fCHO neuron axonal projections (Figure 3B),[82] and the central projections of these neurons in the VNC resemble closely those of the fCHO club neurons and of the tibial chordotonal neurons (Figure 3B).[75,82,83] *86D09-Gal4* femoral expression appeared to be included within that of the large *R46H11-Gal4* club line[78] and to lie beside that of the smaller *R64C04-Gal4* club neuron line (Figure S2A).[75] The neuronal inhibitor driven by *86D09-Gal4* was associated with a striking reduction in female immobility (Figure S2B) during male tremulation (Figure 4A; a decrease of 40% from the *86D09-Gal4* control line and of 31% from the *UAS-TNTE* control line; Table S1). There was no clear effect when the male was not tremulating (Figure 4B) and locomotion of *86D09-Gal4 > UAS-TNTE* females was normal (Figure S2C). Male tremulation was reduced, suggesting that, as on foam, the male may reduce tremulation as a response to low female immobility (Figure S2D). Females expressing the neuronal activator (*UAS-TRPA1*)[84] driven by *86D09-Gal4* became more immobile when the male was not tremulating (Figure 4D). Their immobility during tremulation remained at the normal high level (Figure 4C), and no locomotor defects were observed (Figure S2C). Another line, *R73D10-Gal4*, drives expression in a different subset of 20 fCHO neurons, the "claw" neurons.[75] Expression of the neuronal inhibitor (*UAS-TNTE*) under the control of *R73D10-Gal4* had no effect on mean female immobility during courtship (Figures 4B and S2B) nor on the high level of female immobility during male tremulations (Figure 4A). These results argue that some or all of the ~thirteen *86D09-Gal4*-expressing neurons are club neurons that act specifically in, and are necessary for, the female's response to the male tremulations. A lack of locomotor defects suggests that *86D09-Gal4*-expressing neurons have no role in locomotion as in other insects where neurons in the leg CHOs are specialized to detect vibrations or proprioception.[70,72,85–89] It is not known whether *Drosophila* males also detect substrate-borne vibrations and whether they may use these neurons to do so and to regulate their own tremulation. Our study used behavioral proxies to infer the function of CHO neurons, but further calcium imaging and electrophysiology experiments on single neurons in males and females, ideally during pair courtship, could help understand the thirteen *86D09-Gal4*-expressing neurons further.

Nanchung and Piezo, but not Trpγ, mediate female vibration sensing in 86D09-Gal4-expressing neurons

Finally, we asked which mechanotransducer ion channels, most likely mechanically gated cation channels,[93–96] might mediate vibration sensing in the *86D09-Gal4*-expressing neurons. We tested three genes for cation channels: *nanchung* (*nan*), *dpiezo*, and *transient receptor potential cation channel γ* (*trpγ*), which are expressed in the fCHO;[97–99] Nanchung is involved in mechanosensory transduction;[97,100,101] dPiezo is mechanically activated;[102,103] and Trpγ is required for photomechanosensation, proprioception, and proper gait.[99,104] The roles of these cation channels in leg CHO during fly courtship had not been previously investigated. We verified *nan* and *dpiezo* expression in the leg CHOs using reporter lines and found that they are expressed exclusively in the fCHO, not in the tCHO (Figures S3A and S3B). In previous reports, immunohistochemistry on the central nervous system showed *Nan-Gal4>UAS-mGFP* projections in the gnathal ganglion of the brain, as well as club-shaped projections in the thoracic ganglia of the VNC.[101,105] *dPiezo-Gal4>UAS-mGFP* also displayed expression in the gnathal ganglion of the brain and in club-shaped projections of the thoracic ganglia (Figure S3C; see also Extended Data in Ramdya et al.[98]). These patterns of expression are reminiscent of those observed centrally for fCHO club neurons.[75,82] We knocked down each of these channels in females using *UAS-RNAi* lines under control of *86D09-Gal4* and observed courtship (Figures 4A, 4B, S2B, and S2D). *86D09-Gal4>UAS-nanRNAi* and *86D09-Gal4>UAS-dpiezoRNAi*

first ganglion) enter each thoracic ganglion and bundle together toward the midline of the VNC. The bundles present the club shape typical of club neurons of the fCHO (arrowheads in second and third ganglia).[75,82,83] Scale bars, 100 μm.
(C) The number of cell bodies labeled with GFP was counted in the femoral and tibial chordotonal organs of females carrying the constructs *86D09-Gal4>UAS-mCD8GFP* (5/6 legs among the first pair of legs).

CellPress
OPEN ACCESS

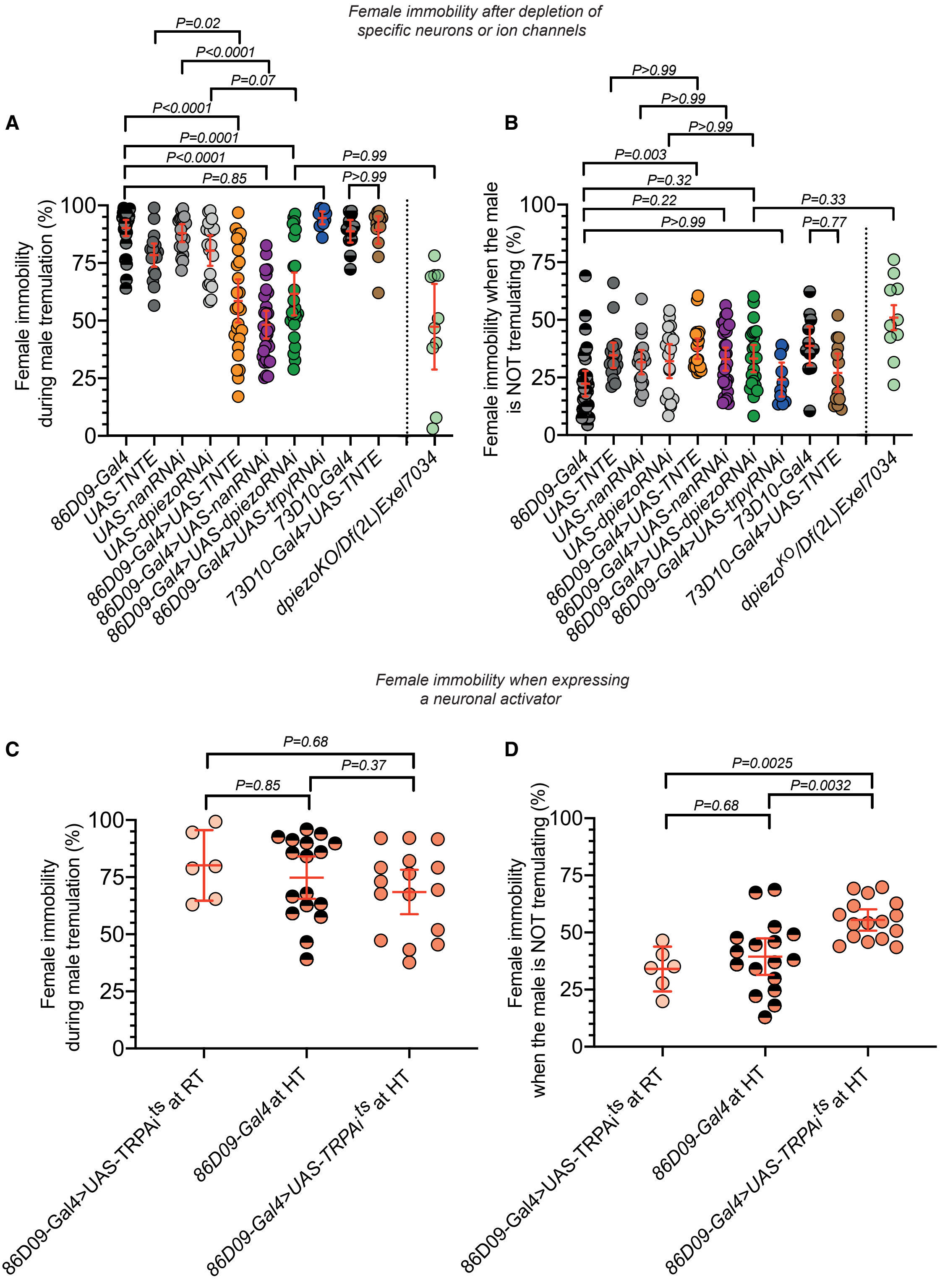

Figure 4. Quantification of experimental and control female immobility during tremulation and during the rest of the courtship in pairs with OrR males

Data for pairs including control females carrying either *86D09*-Gal4 or *73D10-Gal4* (each Gal4 targets different subsets of neurons in the legs) or either the *UAS-TNTE*, *UAS-TRPA1* or one of the *UAS-RNAi* lines and for pairs including experimental females carrying a combination of both a Gal4 and an upstream activating sequence (UAS) and pairs including a *dpiezo*^{KO}/*Df(2L)Exel7034* female. In (A) and (B), ethograms were constructed from analysis of 30, 18, 19, 18, 26, 30, 23, 10, 12, 13, and 10 pairs (in the order illustrated on the graphs).

(legend continued on next page)

females showed a dramatic decrease in total immobility and in immobility coinciding with male tremulations (Figures 4A and S2B). In the *UAS-nanRNAi* control line, female immobility during tremulation was 64% higher than female immobility when the male was not tremulating. But in *86D09-Gal4>UAS-nanRNAi* females, it was only 16% higher, suggesting that female movement becomes less dependent on the tremulations (Figures 4A and 4B; Table S1; Video S2). This was also associated with significantly reduced levels of copulation success (Figure S2G). Females *86D09-Gal4>UAS-dpiezoRNAi* displayed levels of immobility during tremulation that were 36% lower than those of *UAS-dpiezoRNAi* controls (Figure 4A). In addition, *dpiezo*^{KO}*/Df(2L) Exel7034* females, in which *dpiezo* expression was abolished, showed reduced levels of immobility during courtship, and female immobility became independent of the tremulations (Figures 4A, 4B, and S2B; Table S1). *86D09-Gal4>UAS- trpγRNAi* females were unaffected (Figures 4A, 4B, S2B, and S2D). In all three RNAi experiments, climbing and walking trajectories were normal and there were no locomotor defects (Figures S2C, S2D, and S2H).

Together, these results suggest that Nan and dPiezo function in *86D09-Gal4*-expressing neurons to mediate the female's immobility response to tremulations. In flies, Nan, Inactive (Iav), and NompC have been found to work together in the antennal CHO for hearing.[97,100,106] Nan functions with the TRPA channel Waterwitch and with the TRPV channel Iav during hygrosensing.[101] Our findings in the leg CHOs provide an important entry point to investigate the mechanotransducer complex that detects substrate-borne vibrations; the other actors involved in this mechanotransduction remain to be determined, as well as whether Nan and dPiezo act in the same mechanosensory pathway for the reception of the vibrations. Piezo proteins account for most of the gentle touch sensitivity of vertebrates, including vibrations applied to the skin, and they are expressed in the dorsal root ganglion innervating vibration-sensitive cells, such as the Merkel cells and the Meissner's corpuscles.[107–110] Our results and those of other *Drosophila* studies[102,103,111–115] suggest that the roles of Piezo in mechanotransduction (here, for the gentle touch modality of vibration sensation) are diverse and conserved from adult flies to vertebrates.[116]

STAR★METHODS

Detailed methods are provided in the online version of this paper and include the following:

- KEY RESOURCES TABLE
- RESOURCE AVAILABILITY
  - Lead contact
  - Materials availability
  - Data and code availability
- EXPERIMENTAL MODEL AND SUBJECT DETAILS
  - Wild *Drosophila melanogaster*
  - Oregon R, dpiezo mutant flies, all Gal4 and UAS lines during maintenance in the laboratory fly stocks, as well as R64C04-Gal4, R46H11-Gal4, 86D09-Gal4, UASmCD8GFP, R64C04-Gal4 > UASmCD8GFP, 86D09Gal4 > UASmCD8GFP and R46H11-Gal4 > UASmCD8GFP flies used for fluorescent expression
  - 86D09-Gal4, 73D10-Gal4, UAS-dpiezoRNAi, UAS-TNTE, UAS-nanchungRNAi, UAS-trpγRNAi, UAS-TRPA1 and their combined crosses, used for behavioral assays
- METHOD DETAILS
  - Laser vibrometry
  - Behavioral courtship assays
  - Courtship behavior annotations
  - Negative geotaxis climbing assays
  - Principal component analysis
  - Immunohistochemistry and microscopy
- QUANTIFICATION AND STATISTICAL ANALYSIS
  - Laser Vibrometry
  - Courtship Behavior Annotations
  - Negative Geotaxis Climbing Assay
  - Quantification of copulation success
  - Details for each figure

SUPPLEMENTAL INFORMATION

Supplemental information can be found online at https://doi.org/10.1016/j.cub.2021.06.002.

ACKNOWLEDGMENTS

We thank Paul Conduit, Corinne Tovey, Jose Casal, Peter Lawrence, and Jonathan Lee for critical reading of the manuscript; the reviewers for their constructive feedback on our manuscript; Berthold Hedwig for help with the laser vibrometer; and Matt Wayland for help with confocal microscopy. We thank P. Lawrence, B. Hedwig, J. Casal, and P. Conduit for continual support. This reresearch was funded by the Wellcome Trust (grant number 107060/Z/15/Z to P. Lawrence), a Sir Isaac Newton Trust Research Grant (16.24(i) to B. Hedwig and C.C.G.F.), the G.C. Grindley fund (to V.B.P.), the Balfour-Browne Trust fund (to K.M.), and The Department of Zoology and the School

(A) The percentage of time where females were immobile while the male was tremulating is significantly lower only for pairs that included a female *86D09-Gal4>UAS-TNTE*, *86D09-Gal4>UAS-*nanRNAi, or *86D09-Gal4* > *UAS-*dpiezoRNAi. *dpiezo*^{KO}*/Df(2L)Exel7034* females behave similarly to *86D09-Gal4>UAS-*d*piezoRNAi* females.
(B) Same pairs as (A). The percentage of time where females were immobile while the male was not tremulating is 40% lower for *86D09-Gal4* control females compared with *86D09-Gal4>UAS-TNTE* females, but the immobility of the *UAS-TNTE* control line is only 7.5% lower than that of *86D09-Gal4>UAS-TNTE* females; all other pairs behaved similarly to all their associated controls. The immobility of *dpiezo*^{KO}*/Df(2L)Exel7034* females is similar to that of *86D09-Gal4* > *UAS-*d*piezoRNAi* females.
(C) The percentage of time where females were immobile while the male tremulated is shown for pairs filmed at high temperature (HT) (31°C), including control females carrying only the *86D09*-Gal4 construct (n = 17) or *86D09*Gal4 > *UAS-TRPA1* females (n = 16). Data are also shown at room temperature (RT) (23°C) for pairs including females *86D09-Gal4>UAS-TRPA1* (n = 6). Female immobility during male tremulation is similarly high in all 3 types of pairs. This is also the case for total female immobility and male tremulation during courtship (Figures S2E and S2F).
(D) The percentage of time where females were immobile while the male abdomen was not tremulating is shown for the same pairs as (C). *86D09-Gal4>UAS-TRPA1* female immobility at high temperature is significantly higher than controls at RT and HT.
See also Figures S2 and S3, Table S1, and Video S2.

of Biology of the University of Cambridge (to C.C.G.F.). The work benefited from use of the Imaging Facility, Department of Zoology, supported by a Sir Isaac Newton Trust Research Grant (18.07ii(c)). This work is licensed under a Creative Commons Attribution 4.0 International License.

AUTHOR CONTRIBUTIONS

Conceptualization, supervision, validation, project administration, funding acquisition, C.C.G.F.; investigation, formal analysis, C.C.G.F., E.G.Z.M., J.P.G., K.M., V.B.P., L.S., L.E.K., and A.C.; funding acquisition, K.M. and V.B.P.; writing – original draft, C.C.G.F.; writing – review and editing, C.C.G.F. and E.G.Z.M.

DECLARATION OF INTERESTS

The authors declare no competing interests.

INCLUSION AND DIVERSITY

One or more of the authors of this paper self-identifies as a member of the LGBTQ+ community. While citing references scientifically relevant for this work, we also actively worked to promote gender balance in our reference list. The author list of this paper includes contributors from the location where the research was conducted who participated in the data collection, design, analysis, and/or interpretation of the work.

Received: August 20, 2020
Revised: May 11, 2021
Accepted: June 1, 2021
Published: June 25, 2021

REFERENCES

1. Hill, P.S.M. (2009). How do animals use substrate-borne vibrations as an information source? Naturwissenschaften *96*, 1355–1371.
2. Hoch, H., Deckert, J., and Wessel, A. (2006). Vibrational signalling in a Gondwanan relict insect (Hemiptera: Coleorrhyncha: Peloridiidae). Biol. Lett. *2*, 222–224.
3. Cocroft, R.B., and Rodríguez, R.L. (2005). The behavioral ecology of insect vibrational communication. Bioscience *55*, 323–334.
4. Hill, P.S.M. (2008). Vibrational Communication in Animals (Harvard University).
5. Hill, P.S.M. (2001). Vibration and animal communication: a review. Am. Zool. *41*, 1135–1142.
6. Hill, P.S.M., and Wessel, A. (2016). Biotremology. Curr. Biol. *26*, R187–R191.
7. Virant-Doberlet, M., and Čokl, A. (2004). Vibrational communication in insects. Neotrop. Entomol. *33*, 121–134.
8. Fabre, C.C.G., Hedwig, B., Conduit, G., Lawrence, P.A., Goodwin, S.F., and Casal, J. (2012). Substrate-borne vibratory communication during courtship in *Drosophila* melanogaster. Curr. Biol. *22*, 2180–2185.
9. Mazzoni, V., Anfora, G., and Virant-Doberlet, M. (2013). Substrate vibrations during courtship in three *Drosophila* species. PLoS ONE *8*, e80708.
10. Busnel, R.G., Pasquinelly, F., and Dumortier, C. (1955). La trémulation du corps et la transmission aux supports des vibrations en résultant comme moyen d'information à courte portée des Éphippigères femelle et mâle. Ibid. Bull. Soc. Zool. Fr. *80*, 18–22.
11. Medina, I., Casal, J., and Fabre, C.C.G. (2015). Do circadian genes and ambient temperature affect substrate-borne signalling during *Drosophila* courtship? Biol. Open *4*, 1549–1557.
12. Bussell, J.J., Yapici, N., Zhang, S.X., Dickson, B.J., and Vosshall, L.B. (2014). Abdominal-B neurons control *Drosophila* virgin female receptivity. Curr. Biol. *24*, 1584–1595.
13. Markow, T.A., and Hanson, S.J. (1981). Multivariate analysis of *Drosophila* courtship. Proc. Natl. Acad. Sci. USA *78*, 430–434.
14. Shaw, K.L., and Herlihy, D.P. (2000). Acoustic preference functions and song variability in the Hawaiian cricket *Laupala cerasina*. Proc. Biol. Sci. *267*, 577–584.
15. Gerhardt, H.C., Huber, F., and Simmons, A.M. (2003). Acoustic communication in insects and anurans: Common problems and diverse solutions. Physiol. Entomol. *28*, 62–63.
16. Ewing, A.W., and Bennet-Clark, H.C. (1968). The courtship songs of *Drosophila*. Behaviour *31*, 288–301.
17. Doherty, J.A. (1991). Song recognition and localization in the phonotaxis behavior of the field cricket, *Gryllus bimaculatus* (Orthoptera: Gryllidae). J. Comp. Physiol. A *168*, 213–222.
18. Doherty, J.A. (1985). Phonotaxis in the cricket, *Gryllus bimaculatus* DeGeer: comparisons of choice and no-choice paradigms. J. Comp. Physiol. A *157*, 279–289.
19. Doherty, J.A., and Storz, M.M. (1992). Calling song and selective phonotaxis in the field crickets, *Gryllus firmus* and *G. pennsylvanicus* (Orthoptera: Gryllidae). J. Insect Behav. *5*, 555–569.
20. Amrein, H. (2004). Pheromone perception and behavior in *Drosophila*. Curr. Opin. Neurobiol. *14*, 435–442.
21. Aranha, M.M., and Vasconcelos, M.L. (2018). Deciphering *Drosophila* female innate behaviors. Curr. Opin. Neurobiol. *52*, 139–148.
22. Bastock, M., and Manning, A. (1955). The courtship of *Drosophila* melanogaster. Behaviour *8*, 85–111.
23. Ellendersen, B.E., and von Philipsborn, A.C. (2017). Neuronal modulation of *D. melanogaster* sexual behaviour. Curr. Opin. Insect Sci. *24*, 21–28.
24. Greenspan, R.J., and Ferveur, J.F.F. (2000). Courtship in *Drosophila*. Annu. Rev. Genet. *34*, 205–232.
25. Spieth, H.T. (1974). Courtship behavior in Drosophila. Annu. Rev. Entomol. *19*, 385–405.
26. Tauber, E., and Eberl, D.F. (2003). Acoustic communication in *Drosophila*. Behav. Processes *64*, 197–210.
27. Hall, J.C. (1994). The mating of a fly. Science *264*, 1702–1714.
28. Ferveur, J.F. (2010). *Drosophila* female courtship and mating behaviors: sensory signals, genes, neural structures and evolution. Curr. Opin. Neurobiol. *20*, 764–769.
29. Billeter, J.C., Rideout, E.J., Dornan, A.J., and Goodwin, S.F. (2006). Control of male sexual behavior in *Drosophila* by the sex determination pathway. Curr. Biol. *16*, R766–R776.
30. Yamamoto, D., Jallon, J.-M., and Komatsu, A. (1997). Genetic dissection of sexual behavior in *Drosophila melanogaster*. Annu. Rev. Entomol. *42*, 551–585.
31. Rodríguez, R.L., Sullivan, L.M., Snyder, R.L., and Cocroft, R.B. (2008). Host shifts and the beginning of signal divergence. Evolution *62*, 12–20.
32. Mortimer, B. (2017). Biotremology: do physical constraints limit the propagation of vibrational information? Anim. Behav. *130*, 165–174.
33. Michelsen, A., Fink, F., Gogala, M., and Traue, D. (1982). Plants as transmission channels for insect vibrational songs. Behav. Ecol. Sociobiol. *11*, 269–281.
34. Hebets, E.A., Elias, D.O., Mason, A.C., Miller, G.L., and Stratton, G.E. (2008). Substrate-dependent signalling success in the wolf spider, *Schizocosa retrorsa*. Anim. Behav. *75*, 605–615.
35. Elias, D.O., Mason, A.C., and Hoy, R.R. (2004). The effect of substrate on the efficacy of seismic courtship signal transmission in the jumping spider *Habronattus dossenus* (Araneae: Salticidae). J. Exp. Biol. *207*, 4105–4110.
36. Elias, D.O., Mason, A.C., and Hebets, E.A. (2010). A signal-substrate match in the substrate-borne component of a multimodal courtship display. Curr. Zool. *56*, 370–378.
37. Čokl, A., Zorović, M., Žunič, A., and Virant-Doberlet, M. (2005). Tuning of host plants with vibratory songs of *Nezara viridula* L (Heteroptera: Pentatomidae). J. Exp. Biol. *208*, 1481–1488.

38. Čokl, A., and Virant-Doberlet, M. (2003). Communication with substrate-borne signals in small plant-dwelling insects. Annu. Rev. Entomol. *48*, 29–50.

39. Elias, D.O., and Mason, A.C. (2014). The role of wave and substrate heterogeneity in vibratory communication: Practical issues in studying the effect of vibratory environments in communication. In Studying Vibrational Communication. Animal Signals and Communication, *Volume 3*, R.B. Cocroft, M. Gogala, P.S.M. Hill, and A. Wessel, eds. (Springer), pp. 215–247.

40. Hernández, M.V., and Fabre, C.C.G. (2016). The elaborate postural display of courting *Drosophila persimilis* flies produces substrate-borne vibratory signals. J. Insect Behav. *29*, 578–590.

41. Soto-Yéber, L., Soto-Ortiz, J., Godoy, P., and Godoy-Herrera, R. (2018). The behavior of adult Drosophila in the wild. PLoS ONE *13*, e0209917.

42. Markow, T.A. (1987). Behavioral and sensory basis of courtship success in *Drosophila* melanogaster. Proc. Natl. Acad. Sci. USA *84*, 6200–6204.

43. Del Pino, F., Jara, C., Pino, L., and Godoy-Herrera, R. (2014). The neuro-ecology of *Drosophila* pupation behavior. PLoS ONE *9*, e102159.

44. Grosjean, Y., Rytz, R., Farine, J.P., Abuin, L., Cortot, J., Jefferis, G.S.X.E., and Benton, R. (2011). An olfactory receptor for food-derived odours promotes male courtship in *Drosophila*. Nature *478*, 236–240.

45. Yack, J. (2016). Vibrational signaling. In Insect Hearing. Springer Handbook of Auditory Research, *Volume 55*, G. Pollack, A. Mason, A. Popper, and R. Fay, eds. (Springer), pp. 99–123.

46. Röhrig, A., Kirchner, W.H., and Leuthold, R.H. (1999). Vibrational alarm communication in the African fungus-growing termite genus Macrotermes (Isoptera, Termitidae). Insectes Soc. *46*, 71–77.

47. Kirchner, W.H., Broecker, I., and Tautz, J. (1994). Vibrational alarm communication in the damp-wood termite *Zootermopsis nevadensi*s. Physiol. Entomol. *19*, 187–190.

48. Howse, P.E. (1964). An investigation into the mode of action of the subgenual organ in the termite, *Zootermopsis angusticollis* Emerson, and in the cockroach, *Periplaneta americana* L. J. Insect Physiol. *10*, 409–416.

49. Ashby, M.F. (2006). The properties of foams and lattices. Philos. Trans. R. Soc. A Math. Phys. Eng. Sci. *364*, 15–30.

50. Ruiz, A., Naveira, H., and Fontdevila, A. (1985). The evolutionary history of *Drosophila buzzatii*. V. Differential survivorship on *Opuntia* between *D. buzzatii* and *D. serido*. Experientia *41*, 129–131.

51. Markow, T.A., and O'Grady, P. (2008). Reproductive ecology of *Drosophila*. Funct. Ecol. *22*, 747–759.

52. Haouas, S., Carton, Y., Marrakchi, M., and David, J. (1984). Reproductive strategy of *D. buzzati* and *D. melanogaster* associated with the prickly pear of Opuntia in Tunisisa. Oecol. Gener *5*, 175–179.

53. Hrabar, N., Virant-Doberlet, M., and Cokl, A. (2004). Species specificity of male southern green stink bug *Nezara viridula* (L.) reactions to the female calling song. Dong Wu Xue Bao *50*, 566–575.

54. Miklas, N., Stritih, N., Čokl, A., Virant-Doberlet, M., and Renou, M. (2001). The influence of substrate on male responsiveness to the female calling song in *Nezara viridula*. J. Insect Behav. *14*, 313–332.

55. Elias, D.O., Mason, A.C., Maddison, W.P., and Hoy, R.R. (2003). Seismic signals in a courting male jumping spider (Araneae: Salticidae). J. Exp. Biol. *206*, 4029–4039.

56. Žunič, A., Virant-Doberlet, M., and Čokl, A. (2011). Species recognition during substrate-borne communication in *Nezara viridula* (L.) (Pentatomidae: Heteroptera). J. Insect Behav. *24*, 468–487.

57. Žunič, A., Doberlet, M.V., and Čokl, A. (2008). Preference of the southern green stink bug (*Nezara viridula*) males for female calling song parameters. Bull. Insectol. *61*, 183–184.

58. Cocroft, R.B., Hamel, J., Su, Q., and Gibson, J. (2014). Vibrational playback experiments: challenges and solutions. In Studying Vibrational Communication: Animal Signals and Communication, *Volume 3*, R. Cocroft, M. Gogala, P. Hill, and A. Wessel, eds. (Springer), pp. 249–276.

59. Michelsen, A. (2014). Physical aspects of vibrational communication. In Studying Vibrational Communication. Animal Signals and Communication, *Volume 3*, R.B. Cocroft, M. Gogala, P.S.M. Hill, and A. Wessel, eds. (Springer), pp. 199–213.

60. Hill, P.S.M., and Shadley, J.R. (2001). Talking back: sending soil vibration signals to lekking prairie mole cricket males'. Am. Zool. *41*, 1200–1214.

61. Čokl, A., Zorović, M., Žunič, A., and Virant-Doberlet, M. (2005). Tuning of host plants with vibratory songs of Nezara viridula L (Heteroptera: Pentatomidae). J. Exp. Biol. *208*, 1481–1488.

62. Čokl, A. (2008). Stink bug interaction with host plants during communication. J. Insect Physiol. *54*, 1113–1124.

63. McNett, G.D., and Cocroft, R.B. (2008). Host shifts favor vibrational signal divergence in *Enchenopa binotata* treehoppers. Behav. Ecol. *19*, 650–656.

64. Gordon, S.D., and Uetz, G.W. (2011). Multimodal communication of wolf spiders on different substrates: evidence for behavioural plasticity. Anim. Behav. *81*, 367–375.

65. Sullivan-Beckers, L., and Hebets, E.A. (2014). Tactical adjustment of signalling leads to increased mating success and survival. Anim. Behav. *93*, 111–117.

66. Gray, B., Bailey, N.W., Poon, M., and Zuk, M. (2014). Multimodal signal compensation: do field crickets shift sexual signal modality after the loss of acoustic communication? Anim. Behav. *93*, 243–248.

67. Stritih Peljhan, N., and Strauß, J. (2018). The mechanical leg response to vibration stimuli in cave crickets and implications for vibrosensory organ functions. J. Comp. Physiol. A Neuroethol. Sens. Neural Behav. Physiol. *204*, 687–702.

68. Strauß, J., Stritih, N., and Lakes-Harlan, R. (2014). The subgenual organ complex in the cave cricket *Troglophilus neglectus* (Orthoptera: Rhaphidophoridae): comparative innervation and sensory evolution. R. Soc. Open Sci. *1*, 140240.

69. Strauß, J., Lomas, K., and Field, L.H. (2017). The complex tibial organ of the New Zealand ground weta: sensory adaptations for vibrational signal detection. Sci. Rep. *7*, 2031.

70. Stein, W., and Sauer, A.E. (1999). Physiology of vibration-sensitive afferents in the femoral chordotonal organ of the stick insect. J. Comp. Physiol. A *184*, 253–263.

71. Menzel, J.G., and Tautz, J. (1994). Functional morphology of the subgenual organ of the carpenter ant. Tissue Cell *26*, 735–746.

72. Lakes-Harlan, R., and Strauß, J. (2014). Functional morphology and evolutionary diversity of vibration receptors in insects. In Studying Vibrational Communication. Animal Signals and Communication, *vol. 3*, R. Cocroft, M. Gogala, P. Hill, and A. Wessel, eds. (Springer), pp. 277–302.

73. Howse, P.E. (1965). The structure of the subgenual organ and certain other mechanoreceptors of the termite *Zootermopsis angusticollis* (Hagen). Proc. R. Entomol. Soc. Lond., Ser. A Gen. Entomol. *40*, 137–146.

74. Shanbhag, S.R., Singh, K., and Naresh Singh, R. (1992). Ultrastructure of the femoral chordotonal organs and their novel synaptic organization in the legs of *Drosophila melanogaster* Meigen (Diptera : Drosophilidae). Int. J. Insect Morphol. Embryol. *21*, 311–322.

75. Mamiya, A., Gurung, P., and Tuthill, J.C. (2018). Neural coding of leg proprioception in *Drosophila*. Neuron *100*, 636–650.e6.

76. Nottebohm, E., Ramaekers, A., Dambly-Chaudière, C., and Ghysen, A. (1994). The leg of *Drosophila* as a model system for the analysis of neuronal diversity. J. Physiol. Paris *88*, 141–151.

77. Agrawal, S., Dickinson, E.S., Sustar, A., Gurung, P., Shepherd, D., Truman, J.W., and Tuthill, J.C. (2020). Central processing of leg proprioception in *Drosophila*. eLife *9*, e60299.

78. Jenett, A., Rubin, G.M., Ngo, T.T.B., Shepherd, D., Murphy, C., Dionne, H., Pfeiffer, B.D., Cavallaro, A., Hall, D., Jeter, J., et al. (2012). A GAL4-driver line resource for *Drosophila* neurobiology. Cell Rep. *2*, 991–1001.

79. Tsubouchi, A., Caldwell, J.C., and Tracey, W.D. (2012). Dendritic filopodia, Ripped Pocket, NOMPC, and NMDARs contribute to the sense of touch in *Drosophila* larvae. Curr. Biol. *22*, 2124–2134.

80. Sweeney, S.T., Broadie, K., Keane, J., Niemann, H., and O'Kane, C.J. (1995). Targeted expression of tetanus toxin light chain in *Drosophila* specifically eliminates synaptic transmission and causes behavioral defects. Neuron *14*, 341–351.

81. Kamikouchi, A., Inagaki, H.K., Effertz, T., Hendrich, O., Fiala, A., Göpfert, M.C., and Ito, K. (2009). The neural basis of *Drosophila* gravity-sensing and hearing. Nature *458*, 165–171.

82. Tsubouchi, A., Yano, T., Yokoyama, T.K., Murtin, C., Otsuna, H., and Ito, K. (2017). Topological and modality-specific representation of somatosensory information in the fly brain. Science *358*, 615–623.

83. Phillis, R., Statton, D., Caruccio, P., and Murphey, R.K. (1996). Mutations in the 8 kDa dynein light chain gene disrupt sensory axon projections in the *Drosophila* imaginal CNS. Development *122*, 2955–2963.

84. Hamada, F.N., Rosenzweig, M., Kang, K., Pulver, S.R., Ghezzi, A., Jegla, T.J., and Garrity, P.A. (2008). An internal thermal sensor controlling temperature preference in *Drosophila*. Nature *454*, 217–220.

85. Field, L.H., and Matheson, T. (1998). Chordotonal organs of insects. Adv. Insect Physiol. *27*, 1–228.

86. Field, L.H., and Pflüger, H.-J. (1989). The femoral chordotonal organ: a bifunctional orthopteran (*Locusta migratoria*) sense organ? Comp. Biochem. Physiol. Part A. Physiol. *93*, 729–743.

87. BUSchges, A. (1994). The physiology of sensory cells in the ventral scoloparium of the stick insect femoral chordotonal organ. J. Exp. Biol. *189*, 285–292.

88. Kittmann, R., and Schmitz, J. (1992). Functional specialization of the scoloparia of the femoral chordotonal organ in stick insects. J. Exp. Biol. *173*, 91–108.

89. Sauer, A.E., and Stein, W. (1999). Sensorimotor pathways processing vibratory signals from the femoral chordotonal organ of the stick insect. J. Comp. Physiol. A *185*, 21–31.

90. Omoto, J.J., Keleş, M.F., Nguyen, B.M., Bolanos, C., Lovick, J.K., Frye, M.A., and Hartenstein, V. (2017). Visual input to the *Drosophila* central complex by developmentally and functionally distinct neuronal populations. Curr. Biol. *27*, 1098–1110.

91. Wolff, T., Iyer, N.A., and Rubin, G.M. (2015). Neuroarchitecture and neuroanatomy of the Drosophila central complex: A GAL4-based dissection of protocerebral bridge neurons and circuits. J. Comp. Neurol. *523*, 997–1037.

92. Seelig, J.D., and Jayaraman, V. (2015). Neural dynamics for landmark orientation and angular path integration. Nature *521*, 186–191.

93. Vega, J.A., García-Suárez, O., Montaño, J.A., Pardo, B., and Cobo, J.M. (2009). The Meissner and Pacinian sensory corpuscles revisited new data from the last decade. Microsc. Res. Tech. *72*, 299–309.

94. Chalfie, M. (2009). Neurosensory mechanotransduction. Nat. Rev. Mol. Cell Biol. *10*, 44–52.

95. Christensen, A.P., and Corey, D.P. (2007). TRP channels in mechanosensation: direct or indirect activation? Nat. Rev. Neurosci. *8*, 510–521.

96. Syntichaki, P., and Tavernarakis, N. (2004). Genetic models of mechanotransduction: the nematode *Caenorhabditis elegans*. Physiol. Rev. *84*, 1097–1153.

97. Kim, J., Chung, Y.D., Park, D.Y., Choi, S., Shin, D.W., Soh, H., Lee, H.W., Son, W., Yim, J., Park, C.S., et al. (2003). A TRPV family ion channel required for hearing in *Drosophila*. Nature *424*, 81–84.

98. Ramdya, P., Lichocki, P., Cruchet, S., Frisch, L., Tse, W., Floreano, D., and Benton, R. (2015). Mechanosensory interactions drive collective behaviour in *Drosophila*. Nature *519*, 233–236.

99. Akitake, B., Ren, Q., Boiko, N., Ni, J., Sokabe, T., Stockand, J.D., Eaton, B.A., and Montell, C. (2015). Coordination and fine motor control depend on *Drosophila* TRPγ. Nat. Commun. *6*, 7288.

100. Gong, Z., Son, W., Chung, Y.D., Kim, J., Shin, D.W., McClung, C.A., Lee, Y., Lee, H.W., Chang, D.J., Kaang, B.K., et al. (2004). Two interdependent TRPV channel subunits, inactive and Nanchung, mediate hearing in *Drosophila*. J. Neurosci. *24*, 9059–9066.

101. Liu, L., Li, Y., Wang, R., Yin, C., Dong, Q., Hing, H., Kim, C., and Welsh, M.J. (2007). *Drosophila* hygrosensation requires the TRP channels water witch and nanchung. Nature *450*, 294–298.

102. Shao, L., Chung, P., Wong, A., Siwanowicz, I., Kent, C.F., Long, X., and Heberlein, U. (2019). A neural circuit encoding the experience of copulation in female *Drosophila*. Neuron *102*, 1025–1036.e6.

103. Kim, S.E., Coste, B., Chadha, A., Cook, B., and Patapoutian, A. (2012). The role of *Drosophila* Piezo in mechanical nociception. Nature *483*, 209–212.

104. Xu, X.Z.S., Chien, F., Butler, A., Salkoff, L., and Montell, C. (2000). TRPgamma, a Drosophila TRP-related subunit, forms a regulated cation channel with TRPL. Neuron *26*, 647–657.

105. Jourjine, N., Mullaney, B.C., Mann, K., and Scott, K. (2016). Coupled sensing of hunger and thirst signals balances sugar and water consumption. Cell *166*, 855–866.

106. Zhang, W., Yan, Z., Jan, L.Y., and Jan, Y.N. (2013). Sound response mediated by the TRP channels NOMPC, NANCHUNG, and INACTIVE in chordotonal organs of *Drosophila* larvae. Proc. Natl. Acad. Sci. USA *110*, 13612–13617.

107. Zhang, M., Wang, Y., Geng, J., Zhou, S., and Xiao, B. (2019). Mechanically activated Piezo channels mediate touch and suppress acute mechanical pain response in mice. Cell Rep. *26*, 1419–1431.e4.

108. Ranade, S.S., Woo, S.H., Dubin, A.E., Moshourab, R.A., Wetzel, C., Petrus, M., Mathur, J., Bégay, V., Coste, B., Mainquist, J., et al. (2014). Piezo2 is the major transducer of mechanical forces for touch sensation in mice. Nature *516*, 121–125.

109. Chesler, A.T., Szczot, M., Bharucha-Goebel, D., Čeko, M., Donkervoort, S., Laubacher, C., Hayes, L.H., Alter, K., Zampieri, C., Stanley, C., et al. (2016). The role of Piezo2 in human mechanosensation. N. Engl. J. Med. *375*, 1355–1364.

110. Woo, S.H., Ranade, S., Weyer, A.D., Dubin, A.E., Baba, Y., Qiu, Z., Petrus, M., Miyamoto, T., Reddy, K., Lumpkin, E.A., et al. (2014). Piezo2 is required for Merkel-cell mechanotransduction. Nature *509*, 622–626.

111. Zhang, L., Yu, J., Guo, X., Wei, J., Liu, T., and Zhang, W. (2020). Parallel mechanosensory pathways direct oviposition decision-making in *Drosophila*. Curr. Biol. *30*, 3075–3088.e4.

112. He, L., Si, G., Huang, J., Samuel, A.D.T., and Perrimon, N. (2018). Mechanical regulation of stem-cell differentiation by the stretch-activated Piezo channel. Nature *555*, 103–106.

113. Gou, B., Liu, Y., Guntur, A.R., Stern, U., and Yang, C.H. (2014). Mechanosensitive neurons on the internal reproductive tract contribute to egg-laying-induced acetic acid attraction in *Drosophila*. Cell Rep. *9*, 522–530.

114. Wang, P., Jia, Y., Liu, T., Jan, Y.-N., and Zhang, W. (2020). Visceral mechano-sensing neurons control Drosophila feeding by using Piezo as a sensor. Neuron *108*, 640–650.e4.

115. Min, S., Oh, Y., Verma, P., Whitehead, S.C., Yapici, N., Van Vactor, D., Suh, G.S., and Liberles, S. (2021). Control of feeding by Piezo-mediated gut mechanosensation in *Drosophila*. eLife *10*, e63049.

116. Kefauver, J.M., Ward, A.B., and Patapoutian, A. (2020). Discoveries in structure and physiology of mechanically activated ion channels. Nature *587*, 567–576.

117. Markow, T.A., and O'Grady, P. (2006). *Drosophila*: A Guide to Species Identification and Use (Academic).

118. Gemeno, C., Baldo, G., Nieri, R., Valls, J., Alomar, O., and Mazzoni, V. (2015). Substrate-borne vibrational signals in mating communication of *Macrolophus* bugs. J. Insect Behav. *28*, 482–498.

119. Endo, J., Takanashi, T., Mukai, H., and Numata, H. (2019). Egg-cracking vibration as a cue for stink bug siblings to synchronize hatching. Curr. Biol. *29*, 143–148.e2.

120. Prešern, J., Polajnar, J., de Groot, M., Zorović, M., and Virant-Doberlet, M. (2018). On the spot: utilization of directional cues in vibrational communication of a stink bug. Sci. Rep. *8*, 5418.

121. Achenbach, J.D., and Thau, S.A. (1975). Wave Propagation in Elastic Solids (Elsevier Science).

122. Hedwig, B., and Knepper, M. (1992). NEUROLAB, a comprehensive program for the analysis of neurophysiological and behavioural data. J. Neurosci. Methods *45*, 135–148.

123. Tsai, K.T., and Chou, Y.H. (2019). Random walk revisited: quantification and comparative analysis of *Drosophila* walking trajectories. iScience *19*, 1145–1159.

124. Meijering, E., Dzyubachyk, O., and Smal, I. (2012). Methods for cell and particle tracking. Methods Enzymol. *504*, 183–200.

125. Schindelin, J., Arganda-Carreras, I., Frise, E., Kaynig, V., Longair, M., Pietzsch, T., Preibisch, S., Rueden, C., Saalfeld, S., Schmid, B., et al. (2012). Fiji: an open-source platform for biological-image analysis. Nat. Methods *9*, 676–682.

126. RStudio Team (2019). RStudio: integrated development for R (RStudio).

STAR★METHODS

KEY RESOURCES TABLE

REAGENT or RESOURCE	SOURCE	IDENTIFIER
**Antibodies**		
Goat FITC-conjugated anti-GFP	AbCam	Cat# ab6662; RRID: AB_305635
mouse anti-NC82	Hybridoma bank	Cat# nc82; RRID: AB_2314866
anti-mouse Cy5	JacksonImmunoResearch	Cat# 715-175-151; RRID: AB_2340820
**Experimental models: organisms/strains**		
Wild *Drosophila melanogaster*	Collected from Wild (see Experimental model and subject details)	N/A
Drosophila melanogaster Oregon-R	Gift from the Lawrence laboratory, Cambridge	N/A
Drosophila melanogaster dPiezo-Gal4	BDSC 58771 (on chr. II)	FBti0164865
	BDSC 59266 (on chr. III)	FBti0166812
Drosophila melanogaster Nanchung-Gal4	BDSC 24903	FBti0101148
Drosophila melanogaster R86D09-Gal4	BDSC 40459	FBti0139154
Drosophila melanogaster R73D10-Gal4	BDSC 39819	FBti0138087
Drosophila melanogaster R64C04-Gal4	BDSC 39296	FBti0137440
Drosophila melanogaster R46H11-Gal4	BDSC 50284	FBti0136107
Drosophila melanogaster UAS nanchungRNAi	BDSC 53312	FBti0157920
Drosophila melanogaster UAS-trpγRNAi	BDSC 53313	FBti0157921
Drosophila melanogaster UAS-TNTE	BDSC 28837	FBti0038528
Drosophila melanogaster UAS-TRPA1	BDSC 26264	FBti0114502
Drosophila melanogaster UAS-piezoRNAi	NIG-FLY 8486R-1	FBal0267721
Drosophila melanogaster UAS-mCD8-GFP	BDSC 5137	FBti0012685
Drosophila melanogaster $Piezo^{KO}$	BDSC 58770	FBti0147345
Drosophila melanogaster Df(2L)Exel7034	BDSC 7807	FBab0037912
**Software and algorithms**		
Excel Macro to build ethograms from annotated movies	Described in Fabre et al.[8]	Movie-to-ethogram.xlsm available at: https://github.com/CarolineFabre/Excel-Macro-for-ethograms-
R algorithm to analyze overlapping courtship behaviors	Described in Fabre et al.[8]	script_behavior_R available at: https://github.com/CarolineFabre/Script_behaviour.R

RESOURCE AVAILABILITY

Lead contact

Further information and requests for resources and reagents should be directed to and will be fulfilled by the Lead Contact, Caroline C. Fabre (c.c.g.fabre.03@cantab.net)

Materials availability

This study did not generate new unique reagents.

Data and code availability

Excel macros, R code and datasets used to analyze the overlapping behaviors of pairs of flies during courtship are available in the public repository at the following links:

The Excel Macro used to build ethograms from annotated movies is available at: https://github.com/CarolineFabre/Excel-Macro-for-ethograms-.

The R algorithm used to analyze overlapping courtship behaviors script_behavior_R is available at: https://github.com/CarolineFabre/Script_behaviour.R.

Raw data can be found at: https://github.com/CarolineFabre/Raw-data-from-McKelvey-et-al-...Fabre.

EXPERIMENTAL MODEL AND SUBJECT DETAILS

For experiments requiring females without aristae (“*aristae-removed* females”) or without antennnae (“*antennae-removed* females”), aristae or antennae were cut at their base using microscissors upon eclosion, and the flies were kept in tubes for 4 days to allow for recovery and maturation.

For laser vibrometry experiments, wings were removed at collection so as to reduce noise in the recordings.

Before courting, Oregon R individual males or small groups of five to ten virgin females of the appropriate genotype were kept in vials with fresh laboratory food.

Wild *Drosophila melanogaster*

Wild *Drosophila* flies and pupae were collected in San Michele all’Adige (Trentino, Italy) during the International Symposium on Biotremology by means of local fruit baits. After collection, wild *Drosophila* were kept on a mix of fruits from the region, and reared under 12:12 hr light:dark cycle, at 23°C and with 65% humidity. Subsequently, *Drosophila melanogaster* flies were identified morphogically under light CO_2 anesthesia[117] and kept together to reproduce and give rise to progeny.

Oregon R, dpiezo mutant flies, all Gal4 and UAS lines during maintenance in the laboratory fly stocks, as well as R64C04-Gal4, R46H11-Gal4, 86D09-Gal4, UASmCD8GFP, R64C04-Gal4 > UASmCD8GFP, 86D09Gal4 > UASmCD8GFP and R46H11-Gal4 > UASmCD8GFP flies used for fluorescent expression

Flies were reared under 12:12 hr light:dark cycle, at 23°C and with 65% humidity. Virgin female/male progeny were collected upon eclosion from the pupal case and, if used in courtship assays, tested at 4 days-old. *dpiezo* null mutant females were obtained by generating females carrying the *dpiezo* knockout (KO) allele and the deficiency Df(2L)Exel7034 (where the entire dpiezo genomic region is deleted) on each of the homologous chromosomes 2.[103]

86D09-Gal4, 73D10-Gal4, UAS-dpiezoRNAi, UAS-TNTE, UAS-nanchungRNAi, UAS-trpγRNAi, UAS-TRPA1 and their combined crosses, used for behavioral assays

To restrict expression of the UAS to metamorphosis and adulthood we kept all flies at 18°C during embryonic and larval development before moving them to room temperature (23°C). Control flies were subjected to the same conditions as experimental flies preceding pair assays.

UAS-TNTE (Tetanus toxin) specifically cleaves neuronal Synaptobrevin, which is essential for synaptic vesicle release and neurotransmitter release, and its expression leads to impairment of neuronal functions.[80]

UAS-TRPA1 is a thermosensitive tool and its expression leads to activation of neurons at elevated temperatures because TRPA1 is a heat-activated nonselective cation channel.[84]

METHOD DETAILS

Laser vibrometry

Laser vibrometry of courting pairs were performed at a temperature of around 23°C. Video and laser vibrometer recordings were conducted on a vibration-damped table in a soundproof room. Virgin female/male progeny from wild-caught males and females were collected upon eclosion from the pupal case and directly filmed while courting within 1 week of collection so as not to habituate the wild stock to lab conditions. In all other cases, flies were filmed and courted when 4 days old upon eclosion from the pupal case.

Flies were filmed with a Stingray F-33B camera (Allied Vision) on either the recording membrane, other artificial substrates, or on a sample of fruit or plant. All natural and artificial substrates were prepared with similar thickness (around 5mm) and size (around 10mm × 9mm). To record, the beam of a laser Doppler vibrometer (Polytec OFV 5000 controller, OFV 534 sensor head; Waldbronn, Germany) was directed perpendicular to the surface of a square of reflective tape (3M, 0.5mm^2; Scotchlite, Neuss, Germany) placed in the center onto the surface of the fruit or artificial substrates. Reflective tapes are commonly used in biotremology studies that involve laser vibrometry on non-reflective substrates;[55,118–120] the physics of mechanical waves allows for their energy to be transferred from one solid (i.e., the substrate) to another (i.e., the tape) by wave motion.[121] Signals were digitised with 12bit amplitude resolution with a PCI MIO-16-E4 card (Analog Devices; Norwood, MA) and with LabView (National Instruments; Austin, TX) on a PC. Signals were transformed into .wav data with Cool Edit Pro (Adobe Systems) or Neurolab software.[122] Video and laser vibrometer recordings were synchronized at the start by brief interruption of the laser path; this produces both a momentary peak in the oscillogram and a black frame in the video.

Behavioral courtship assays

Video-imaging of courting pairs were performed at a temperature of around 23°C. Male–virgin female fly pairs were tested at 4 days old. Their behavior was recorded with a 100mm macro lens and a Stingray F-033B camera (Allied Vision Technologies; Stadtroda, Germany) and acquired with the Debut Video Capture (Pro Edition) software into a iMac computer. Apart from experiments on substrates, courting pairs were filmed in transparent plexiglass chambers (10mm diameter and 6 mm height) as in Fabre et al.[8] For experiments on substrates, the substrates used were ripened fruits: apple, banana, prickly-pear; layer of bark from an apple tree; gravel stone; an isolating foam (open-cell type foam characterized by low conductivity and produced for thermal insulation[49]). The foam selected did not appear to produce repellent artificial smells to the flies as courtship index was high (not shown). Cylindrical holes (around 10mm diameter and 9mm depth) were carved within fruit and foam substrates. We also filmed flies on our original recording membrane; a thermal foil, made of silver metallized polyester material, with an albedo of approximately 0.8 (Sub Zero Technology; Leicester, UK). A piece of transparent plexiglass was placed between the flies and the camera to contain the flies. Movies were only taken into account for analysis if the flies spent more than 95% of the time on the substrate as opposed to the plexiglass. Recording was started at the initiation of courtship and for approximately 600 s, or until copulation occurred. Each pair was tested only once.

Courtship behavior annotations

Movies were annotated with semi-automated Annotation software (Peter Brodsky, version 1.3) while watched on a large 27-inch desktop screen, thus allowing behaviors to be detected with great sensitivity and accuracy. We registered male courting behaviors such as orientation toward the female, tremulation of the abdomen (i.e., rapid up-and-down movements of the abdomen, as described in Fabre et al.[8]), extension and vibration of the wings, and also whether the female was moving or stationary (stationary being defined as the female not walking in any direction). Annotations were performed in a randomized and blind manner (movie files were randomly numbered so that the identity of the pair annotated was not identified), and approximately 10% of the annotations were performed twice for comparison (i.e., by two different annotators), with the resulting consensus annotation used when necessary. Each behavior was annotated independently from the others. For experiments testing substrates, annotation was only recorded when both animals were localized on the substrate (instances when either or both animals were localized on the transparent plexiglass top were not included in the analysis). Note, when monitoring abdominal tremulations and female immobility it is very difficult to judge whether the tremulations start just before or just after female stopping, as the tremulations and stopping occur almost simultaneously.

Negative geotaxis climbing assays

Virgin females (aged 4 days old) were inserted into a capped graded tube. The flies were tapped to the bottom and the number of flies crossing a target line localized 4 cm above the bottom of the tube was recorded using a camera.

Principal component analysis

11 walking variables (similar to variables used in Tsai and Chou[123]) were used to perform principal component analysis (PCA) on 4-7 day old individual female flies carrying the constructs *86D09-Gal4, UAS-dpiezoRNAi, UAS-nanRNAi, 86D09-Gal4 > UAS-dpiezoRNAi* or *86D09-Gal4 > UAS-nanRNAi,* and filmed walking for 30 minutes in Petri dishes (diameter 5cms) using a webcam (Logitech); Debut Video Capture (Pro Edition) software was used with an iMac computer for video acquisition. Tracking of fly and analysis of locomotion was performed using the plugin MtrackJ[124] in Fiji.[125] The variables used for PCA in each 5 s time bin were: average and standard deviation of speed, average orientation, average angular velocity, average and standard deviation of horizontal velocity, average and standard deviation of vertical velocity, straightness, magnitude of displacement, weighted average of orientations by distances. Data were standardized prior to computation using the Pearson Correlation treatment. Standardized values were then subjected to PCA[123] using XLSTAT software in Excel (Data Analysis and Statistical Solution for Microsoft Excel, Addinsoft, Paris, France 2017).

Immunohistochemistry and microscopy

In preparation for dissection under the binocular microscope, flies were anaesthetised and placed in a Petri dish with phosphate-buffered saline (PBS) where brain and ventral nerve cords were dissected out of the cuticle, fixed (4% paraformaldehyde, Electron

Microscopy Science) and stained. The following antibodies were used: Goat FITC-conjugated anti-GFP (1:1000, Abcam), mouse anti-NC82 (1:20, Hybridoma bank), anti-mouse Cy5 (1:1000; JacksonImmunoResearch). Samples were mounted in Fluoromount mounting medium (SigmaAldrich) with the anterior part of the brains and ventral side of the ventral nerve cords oriented upward. The samples were imaged with a confocal microscope (Leica SP5) run by LAS AF software. Legs were dissected out and mounted with Fluoromount for confocal imaging or for imaging on a Leica DMi8 microscope mounted with a BSI express camera (Teledyne Photometrics). The software Fiji[125] was used to process the .lif and .tif files. To compare the expression driven by several Gal4 driver lines, we used Fiji to overlay the images based on morphological landmarks (femur-coxa joint, femur-tibia joint, shape of the cuticle).

QUANTIFICATION AND STATISTICAL ANALYSIS

Statistical details of experiments can be found below and in figures. $p < 0.05$ is the threshold of significance. On all graphs, red bars are used to indicate mean and 95% confidence intervals.

Laser Vibrometry

Oscillograms were analyzed with Amadeus Pro (HairerSoft) and Raven Pro (The Cornell Lab of Ornithology, Bioacoustics Research Program) software. Frequency analysis on different substrates was performed using Amadeus Pro (HairerSoft) on 1-3 pulses. The values in decibels (dB) obtained in the y axis were scaled relative to the minimum laser output, with −100 dB corresponding to the minimum laser output velocity of 0.04 μm/s and the amplitudes measured increasing relative to this minimal value. The repetition rate of a vibratory signal is defined as the number of pulses per second, i.e., 1/IPI, converted in Herz (Hz).

Courtship Behavior Annotations

Data for each annotated movie were imported into Excel and into Rstudio [147]. As in [9], the resulting file obtained for each annotated movie showed for each period of 1 s, whether the male was moving or stationnary, and whether the male abdomen was tremulating or not (or, in Figure S1B, whether the male wing was fluttering or not). For each movie we calculated the percentage of time that the female was immobile when the male was tremulating its abdomen (or, in Figure S1B, when the male wing was fluttering). We generated the plots using Prism (GraphPad). For statistical analysis and generation of diagrams, we used Microsoft Excel macros, the R programming language and software environment[126] and Prism (GraphPad).

Negative Geotaxis Climbing Assay

The percentage of females having passed the threshold line after 3 s is represented. Each climbing assay tested around 20 flies.

Quantification of copulation success

Mating was considered and represented as successful if male and female copulated within 10 minutes of courtship.

Details for each figure

Figure 1: Dunnett's T3 multiple comparisons tests were used to calculate p values that can be found in the figure and in the figure legend. *n*-numbers are given in figure legend and represent number of video clips; *n*-numbers are given in figure legend; each video clip is made from one unique courtship pair. Each data point corresponds to the data from one video clip. In figure legend, averages are given as the mean ± standard error of the mean.

Figure 2A: The y axis shows the relative amplitude of the signals (in decibels, on a logarithmic scale) and the x axis shows increasing frequencies in Herz. Vertical black lines are present if the frequency was recorded in the signal. Analysis of pulses was performed using Amadeus Pro (hairerSoft).

Figure 2B: Dunnett's T3 multiple comparisons test was used to calculate p values that can be found in the figure. *n*-numbers are given in figure legend and represent number of pulses recorded across multiple courtship pairs (see legend). Each interpulse interval (IPI) value is represented by a circle on the graph.

Figure 4: Dunnett's T3 multiple comparisons tests were used to calculate p values that can be found in the figure. *n*-numbers are given in figure legend and represent number of video clips; each video clip (each circle) is made from one unique courtship pair.

Figures S1A and S1B: Two-tailed unpaired t test was used to calculate p values that can be found in the figure legend. *n*-numbers are given in the figure and represent number of video clips; each video clip is made from one unique courtship pair. In figure legend, averages are given as the mean ± standard error of the mean.

Figure S1C: Dunnett's T3 multiple comparisons tests were used to calculate p values that can be found in the figure. *n*-numbers are given in figure legend and represent number of video clips; each video clip is made from one unique courtship pair.

Figure S1D: Two-tailed unpaired t test was used to calculate p values that can be found in the figure. n-numbers are given in figure legend and represent number of pulses recorded across multiple courtship pairs (see figure legend). Each pulse is represented by a circle on the graph.

Figure S1E: Two-tailed unpaired t test was used to calculate p values that can be found in the figure. *n*-numbers are given in the figure and represent number of video clips; each video clip is made from one unique courtship pair.

Figure S1F: Dunnet's T3 multiple comparison tests was used to calculate p values that can be found in the figure. For *n*-numbers see legend of Figures 1D–1F.

CellPress
OPEN ACCESS

Figures S2B and S2D: Dunnett's T3 multicomparison tests were used to calculate p values that can be found in the figure. *n*-numbers can be found in legend of Figures 4A and 4B and represent number of video clips (each video clip is made from one unique courtship pair).

Figure S2C: Dunnett's T3 multicomparison tests were used to calculate p values that can be found in the figure. n-number can be found in figure legend and represent the number of climbing assays performed (each circle represent one climbing assay – see sections above for method and below for statistical details).

Figures S2E and S2F: Dunnett's T3 multiple comparisons tests were used to calculate p values that can be found in the figure. n-number can be found in legend of Figures 4C and 4D.

Figure S2G: Dunnett's T3 multiple comparisons test was used to calculate p values that can be found in the figure. n-number can be found in figure legend.

Table S1: Dunnett's T3 multiple comparisons test was used to calculate p values that can be found in the figure.

Current Biology, Volume *31*

Supplemental Information

Drosophila females receive male substrate-borne signals through specific leg neurons during courtship

Eleanor G.Z. McKelvey, James P. Gyles, Kyle Michie, Violeta Barquín Pancorbo, Louisa Sober, Laura E. Kruszewski, Alice Chan, and Caroline C.G. Fabre

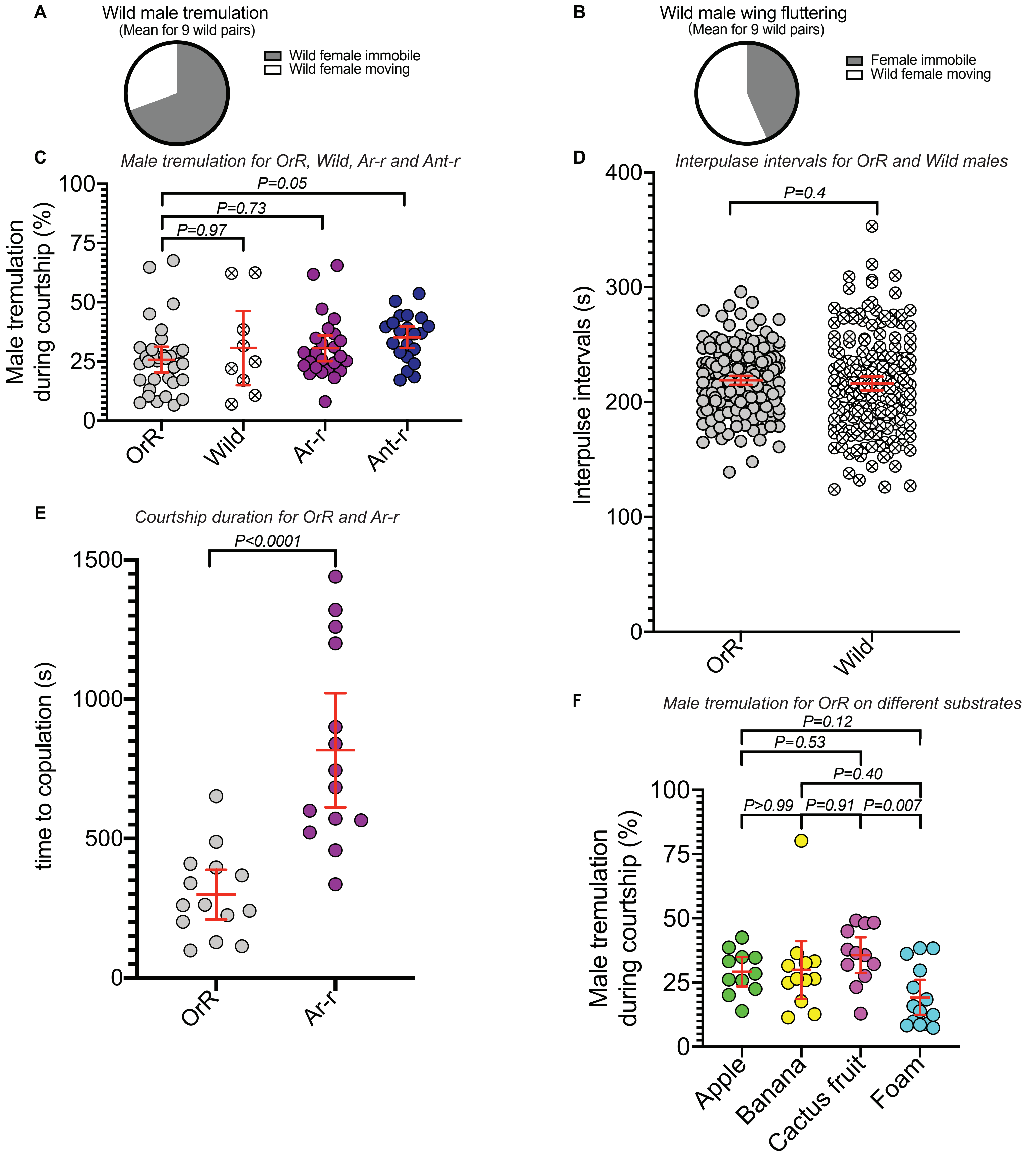

Figure S1. Details of the courtship of wild, OregonR, Ar-r and Ant-r *Drosophila* pairs, related to Figure 1. (A) and (B) compares quantification of the wild female movement with respect to whether the wild male is fluttering his wing or tremulating his abdomen during courtship (wild flies were collected in Italy, see STAR methods). (A) The pie chart shows the percentage of time where wild females were immobile or moving during wild male tremulations. Wild females spent on average 70 ± 7 % of the time being immobile during wild male tremulations. This is significantly higher than the time they spent immobile when the male was not tremulating (46 ± 7 %; see Figure 1C; P=0.03). (B) The pie chart shows the percentage of time where wild females were immobile or moving during wild male wing fluttering (producing the 'love song'). Wild females spent around half of the time being immobile (56 ± 8 %) and half of the time moving during wild male wing fluttering. Wild male wing fluttering did not correlate strongly with female immobility (P=0.46), but wild male tremulations did, confirming findings with laboratory stocks [S1,S2]. (C) shows the total percentage of time where males were tremulating during courtship. This percentage in wild flies (n=9) and Ar-r pairs (n=25) is similar to that of intact OrR pairs (n=32); it is slightly higher in Ant-r pairs (n=22). (D) Interpulse intervals (in seconds) of the substrate-borne vibrations generated by tremulations of laboratory-stock OrR (n=188 pulses recorded) and of wild males (n=188 pulses recorded) recorded on the artificial foil during courtship with OrR females and wild females, respectively. Scatter plots are shown for 3 individu-als for each type. There were no significant differences in the mean interpulse interval between both. (E) shows the duration of court-ship preceding copulation for OrR pairs and for Ar-r pairs. It is significantly increased for Ar-r pairs presumably because *aristae-removed* females cannot receive the species-specific love song that promotes copulation. (F) shows the total percentage of time where OrR males were tremulating during courtship on natural and foam substrates for the same pairs as in Figure 1D-F. This percentage is similar on most substrates, but it differs significantly between cactus fruit and foam.

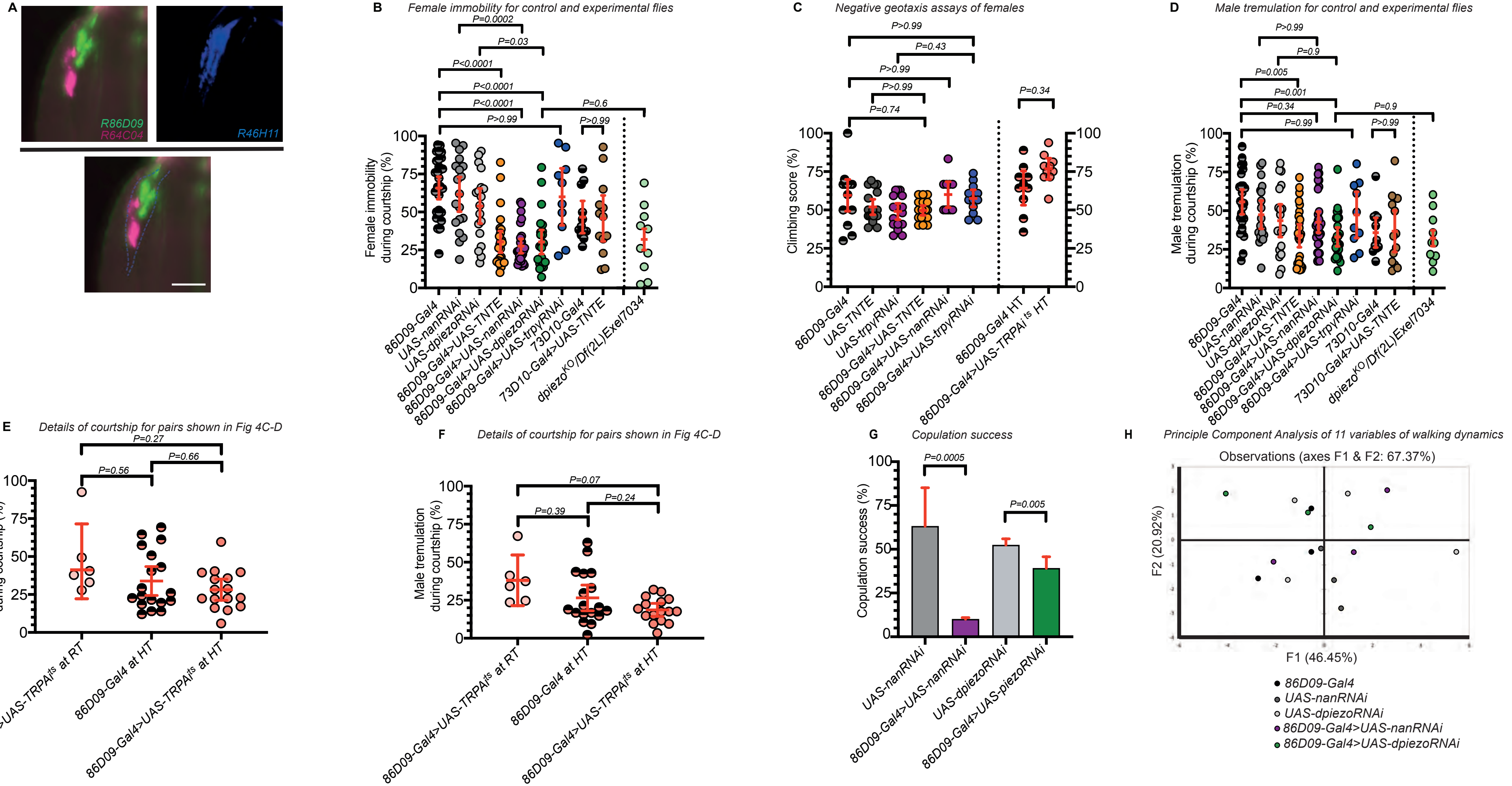

Figure S2. Details of expression, courtship and locomotor assays for experimental and control flies, related to Figure 4. (A) shows side view of three overlaid female legs (microscope z-stack projections); (left) shows overlaid expression of GFP under the control of either *R64C04-Gal4* (magenta; this line drives expression in around 30 club neurons[S3]) or *86D09-Gal4* (green); (right) shows expression of GFP under the control of *R46H11-Gal4* (blue; this line drives expression in a large portion of the club neurons[S4]); (bottom) shows overlay of the expression patterns for the three Gal4 lines (*R46H11-Gal4* expression is indicated using blue dashed lines); scale bar, 40µm (B) shows quantification of female immobility during courtship for pairs including control females carrying either *86D09-Gal4* or *73D10-Gal4*, or one of the UAS-RNAi lines, or a combination of both a Gal4 and a UAS, and pairs including a *dpiezo*^{KO}*/Df(2L)Exel7034* female. Ethograms were constructed from the same pairs shown in Figure 4A-B. Note that females carrying Gal4 and UAS construct show levels of immobility that differ from OrR females during courtship. We have no explanation for this phenomenon, which we have observed in our experimental set-up with all engineered lines carrying Gal4/UAS that we tested. *86D09-Gal4>UAS-TNTE*, *86D09-Gal4>UAS-nanRNAi*, *86D09-Gal4>UAS-dpiezoRNAi* all show significantly lower mean female immobility in comparison to their associated controls. *dpiezo*^{KO}*/Df(2L)Exel7034* females behaved similarly to *86D09-Gal4>UAS-dpiezoRNAi* females. *86D09-Gal4>UAS-trpγRNAi* females have similar immobility to their associated control. The mean value obtained for the immobility of *73D10-Gal4>UAS-TNTE* females is unchanged compared with *73D10-Gal4* control females. (C) Negative geotaxis climbing assays of females carrying a Gal4 or a UAS alone or in combination, using a tap down assay. Climbing scores were obtained from analysis of 14, 18, 17, 19, 19, 13, 10 and 10 climbing assays (in the order illustrated on the graph). Assays displayed to the right of the dashed line were performed at high temperature. No climbing defect could be observed in any of the females tested. Note that adult knockouts for the gene *dpiezo* have previously been shown to have a normal locomotion in climbing assays[S5]. (D) shows the total percentage of time where males were tremulating during courtship for the same pairs as in (A) and Figure 4A-B. Males tremulate less when paired with females *86D09-Gal4>UAS-TNTE* and *86D09-Gal4>UAS-dpiezoRNAi*. In all other pairs males displayed similar level of tremulations to associated controls. (E) shows quantification of experimental and control female immobility during courtship in the same conditions and for the same pairs as in Figure 4C-D. Female immobility during courtship is similar in all 3 types of pairs. (F) shows the total percentage of time where males were tremulating during courtship for the same pairs as in (D) and Figure 4C-D. This percentage is similar in all 3 types of pairs. Note that males tremulate at a different frequency than that observed for males in OrR pairs, which we suppose is a response to the level of immobility and receptivity of females carrying the constructs (see B), courtship being an interactive behaviour between mates. (G) The percentage of copulation success is shown for pairs including control females carrying either *UAS-nanRNAi* (n=19) or *UAS-dpiezoRNAi* (n=21), and experimental females carrying the combination of *86D09-Gal4* with one of these *UAS-RNAi* (n=30 and n=23, respectively). This percentage is significantly lower in pairs including the experimental flies, suggesting that interfering with these ion channels in *86D09-Gal4*-neurons also altered mating. (H) Principle Component Analysis (PCA) of 11 variables of walking dynamics measured in a walking assay and comparing across females carrying either *86D09-Gal4*, *UAS-nanRNAi, UAS-dpiezoRNAi*, *86D09-Gal4>UAS-dpiezoRNAi* or *86D09-Gal4>UAS-nanRNAi*. 3/4 flies were tracked for each genotype and each dot represents the data point for a specific fly. The PCA shows 67.37% of the total variability in the data set. There is no obvious clustering of the data points, which suggests that locomotion does not vary depending on the constructs present in the female. Differences display solely variations between flies, independently of their genotype.

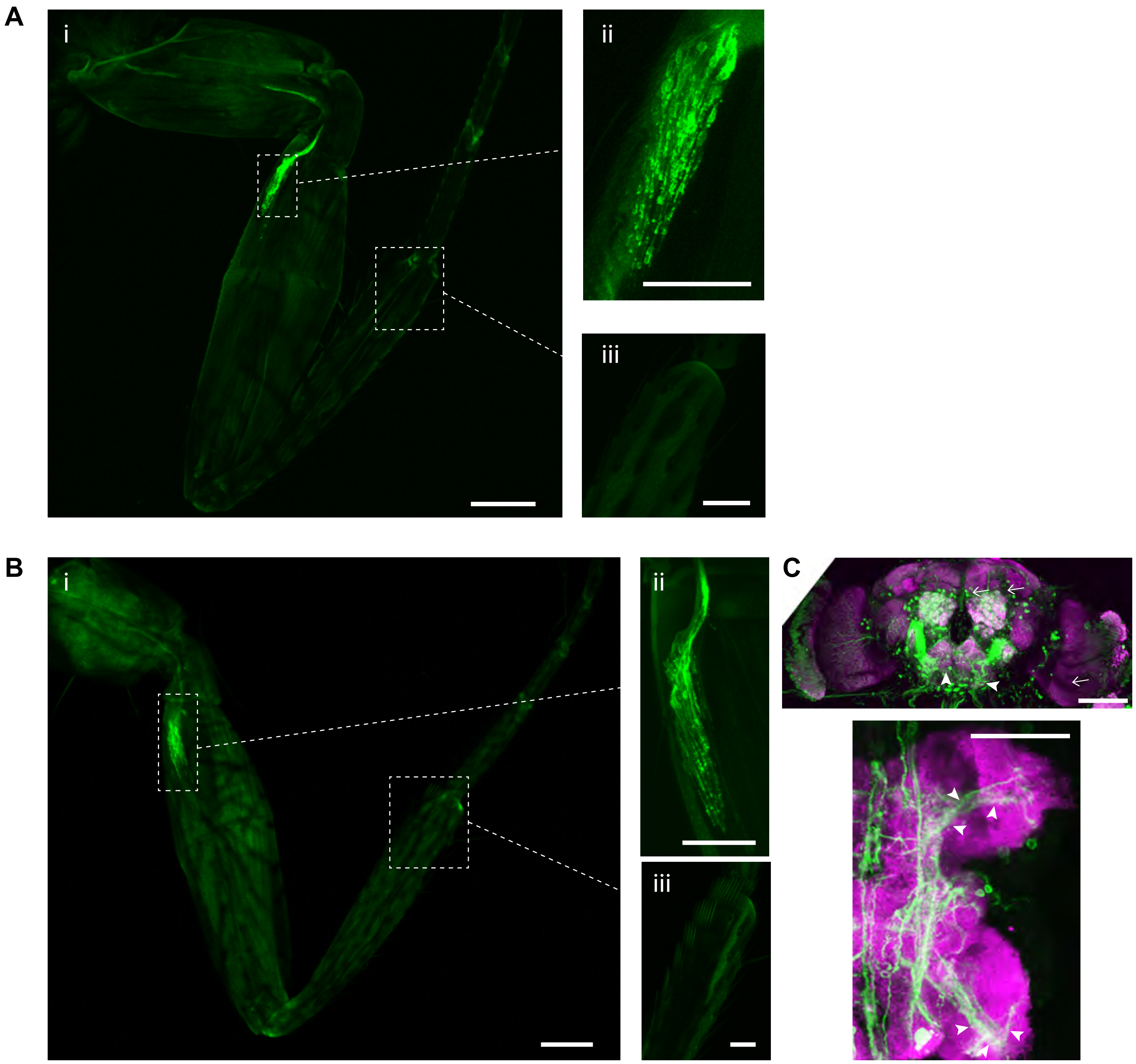

Figure S3. Pattern of expression of the Gal4 lines *Nan-Gal4* and *dPiezo-Gal4* in the leg and in the central nervous system, related to Figure 4. (A) A confocal image of the front leg of a female carrying the construct *Nan-Gal4>UASmCD8GFP.* (i) shows expression (bright green) in the femoral chordotonal organ and no expression in the tibial chordotonal organ; the axons of the femoral chordotonal neurons bundle to project upwards towards the trochanter and the central nervous system. Scale bar indicates 100 µm; (ii) shows expression in hundreds of cell bodies and processes of the femoral chordotonal organ (bright green). Scale bar indicates 40 µm; (iii) shows no expression where the tibial chordotonal organ is localised. Scale bar indicates 40 µm. Light green is autofluorescence from the cuticle. Note that, in[S6,], the adult brain of *Nan-Gal4>UAS-mCD8GFP* does not display GFP in the optic lobes nor in the ellipsoid body or ring neurons where *86D09-Gal4* also displayed some expression. (B) A confocal image of the front leg of a female carrying the construct *dPiezo-Gal4>UAS-mCD8GFP*. (i) shows expression (bright green) in the femoral chordotonal organ and no expression in the tibial chordotonal organ. Scale bar indicates 100 µm; (ii) shows expression in hundreds of cell bodies and processes of the femoral chordotonal organ (bright green). The axons of the neurons bundle to project upwards towards the trochanter and the central nervous system. Scale bar indicates 40 µm; (iii) shows no expression where the tibial chordotonal organ is localised. Scale bar indicates 40 µm. Light green is autofluorescence from the cuticle. (C) A confocal image showing axon terminals (green) of *dPiezo-Gal4>UAS-mCD8GFP*-expressing neurons in a female fly brain (top) and ventral nerve cord (VNC, bottom) labelled by the neuropil marker NC82 (magenta). (top) In the brain small processes targeting the anterior side of the brain can be distinguished in the gnathal ganglion (arrowheads). They resemble descriptions of some femoral chordotonal neuron projections that target directly this region of the brain[S8]. GFP is not visible in the optic lobes nor in the ellipsoid body or ring neurons where *86D09-Gal4* also displayed some expression (arrows; and see Extended Data in[S8]); (bottom) In the ventral nerve cord (only the first two hemi-neuropils are displayed), projections display the characteristic club shape of femoral chordotonal neuron projections (arrowheads); they are localised in the most dorsal layer in the middle of the neuropil of the thoracic ganglia that is also characteristic of the fCHO club neurons of the femoral chordotonal organ[S3,S9]. Scale bars indicate 100 µm. Note, *dPiezo-Gal4*[S5] may not perfectly represent *dpiezo* expression as it does not include the complete endogeneous promoter but includes only 1000bp of upstream promoter sequence[S5]; its expression pattern in the adult brain appears, in general, similar to the expression pattern of a recently made *Trojan-dpiezo-Gal4* line (http://flypush.imgen.bcm.tmc.edu/pscreen/rmce/rmce.php?entry=RM00971), suggesting that the expression pattern of *dpiezo-Gal4*[S5] is broadly reflective of endogenous *dpiezo* expression. Thus, while we cannot completely rule out that endogenous *dpiezo* could be expressed within brain neurons targeted by *86D09-Gal4*, the most parsimonious explanation for our results presented in Figure 4 is that it is the depletion of *dpiezo* within the *86D09-Gal4* fCHO neurons, rather than its depletion within the *86D09-Gal4*-expressing brain neurons, that leads to the observed phenotype.

**Comparison of female immobility for OrR, Ar-r and Ant-r pairs depending on whether the male is tremulating or not**					
Dunnett's T3 multiple comparisons test	Mean Diff.	95.00% CI of diff.	Significant?	Summary	Adjusted P Value
IT OrR vs. INT OrR	26.96	11.36 to 42.56	Yes	****	<0.0001
IT Ar-r vs. INT Ar-r	15.46	-0.4716 to 31.39	Yes	*	0.0338
IT Ant-r vs. INT Ant-r	41.27	25.23 to 57.31	Yes	****	<0.0001
**Comparison of female immobility on different substrates depending on whether the male is tremulating or not**					
Dunnett's T3 multiple comparisons test	Mean Diff.	95.00% CI of diff.	Significant?	Summary	Adjusted P Value
IT Apple vs. INT Apple	14.25	-2.991 to 31.48	No	ns	0.1056
IT Banana vs. INT Banana	23.8	-1.102 to 48.69	Yes	*	0.0367
IT Cactus fruit vs. INT Cactus fruit	18.82	-0.2839 to 37.92	Yes	*	0.0285
IT Foam vs. INT Foam	-44.66	-74.23 to -15.09	Yes	***	0.0003
**Comparison of female immobility in control and experimental pairs depending on whether the male is tremulating or not**					
Dunnett's T3 multiple comparisons test	Mean Diff.	95.00% CI of diff.	Significant?	Summary	Adjusted P Value
IT 86D09-Gal4 vs. *INT 86D09-Gal4*	67.89	54.67 to 81.12	Yes	****	<0.0001
IT UAS-TNTE vs. *INT UAS-TNTE*	43.77	28.70 to 58.85	Yes	****	<0.0001
IT UAS-nanRNAi vs. *INT UAS-nanRNAi*	56.32	43.06 to 69.59	Yes	****	<0.0001
IT UAS-piezoRNAi vs. *INT UAS-piezoRNAi*	48.35	28.33 to 68.37	Yes	****	<0.0001
IT 86D09-Gal4>UAS-TNTE vs. *INT 86D09-Gal4>UAS-TNTE*	21.06	-0.4707 to 42.58	Yes	*	0.03
IT 86D09-Gal4>UAS-NanRNAi vs. *INT 86D09-Gal4>UAS-nanRNAi*	15.26	-0.6176 to 31.15	Yes	*	0.04
IT 86D09-Gal4>UAS-piezoRNAi vs. *INT 86D09-Gal4>UAS-piezoRNAi*	28.38	5.534 to 51.22	Yes	**	0.001
IT 86D09-Gal4>UAS-trpγRNAi vs. *INT 86D09-Gal4>UAS-trpγRNAi*	64.24	49.30 to 79.18	Yes	****	<0.0001
IT 73D10-Gal4 vs. *INT 73D10-Gal4*	50.09	29.03 to 71.16	Yes	****	<0.0001
IT 73D10-Gal4>UAS-TNTE vs. *INT 73D10-Gal4>UAS-TNTE*	62.32	40.71 to 83.92	Yes	****	<0.0001
IT *dpiezo*^{KO}*/Df(2L)Exel7034* vs. INT*dpiezo*^{KO}*/Df(2L)Exel7034*	-3.588	-51.61 to 44.43	No	ns	>0.9999

Table S1. Comparison of female immobility during courtship depending on whether the male is tremulating or not, related to Figures 1 and 4. In the first column, IT stands for "Immobility during male Tremulation", and INT for "Immobility when the male is Not Tremulating". The **top panel** shows statistical data for OrR, Ar-r and Ant-r pairs. A Dunnett's T3 multiple comparisons test was applied to values displayed in figure 1B and 1C, to verify if female immobility varied significantly whether the male tremulated or not. P values are presented in the table. They show that female immobility is significantly different in the OrR, Ar-r and Ant-r pairs depending on whether the male tremulates or not. In the 3 types of pairs the female is significantly more immobile when the male tremulates. The **middle panel** shows statistical comparison of female immobility on different substrates depending whether the male is tremulating or not. A Dunnett's T3 multiple comparisons test was applied to values displayed in figure 1E and 1F, to verify if female immobility on one substrate varied significantly whether the male tremulated or not. P values show that female immobility during tremulation is significantly different on the banana, cactus fruit and foam compared to immobility when the male is not tremulating. The **bottom panel** shows statistical data using a Dunnett's T3 multiple comparisons test applied to values displayed in figure 4A and 4B, to verify if control and experimental female immobility varied significantly whether the male tremulated or not. P values show that female immobility is significantly different depending on whether the male tremulates or not (female is significantly more immobile when the male tremulates), but less so when the females are carrying the constructs *86D09-Gal4>UAS-TNTE*, *86D09-Gal4>UAS-nanRNAi* or *86D09-Gal4>UAS-dpiezoRNAi.* This suggests that the movement of these three types of females is tending towards being independent of the tremulations. The immobility of females *dpiezo*^{KO}*/Df(2L)Exel7034* is similar, whether the male is tremulating or not.

Substrate	Fly falling on the substrate from the opposite side of the chamber	Fly walking on the substrate	Substrate-borne vibrations generated by tremulations	Wing fluttering substrate-borne components	Number of flies recorded
Stone	+	-	-	-	5
Wood slice	+	-	-	-	3
Banana	+	+	+	+	10
Apple	+	+	+	+	10
Prickly Pear Cactus fruit	+	+	+	+	15
Foil	+	+	+	+	20
Isolating Foam	-	-	-	-	3

Table S2. Types of signals picked up by the laser doppler vibrometer during *D. melanogaster* OrR courtship on different substrates, related to Figure 2. In all cases the beam of the laser was targeted onto the substrate around the middle of the courtship area of similar size and substrate thickness (see STAR methods). “Wing fluttering substrate-borne components” refers to the signals generated by wing fluttering in the substrate recently described by[S10]. The + indicates that signal was picked up by the laser, - indicates that no signal was visible on the recordings.

Supplemental References

S1. Fabre, C.C.G., Hedwig, B., Conduit, G., Lawrence, P.A., Goodwin, S.F., and Casal, J. (2012). Substrate-borne vibratory communication during courtship in *Drosophila* melanogaster. Curr. Biol. *22*, 2180–2185.

S2. Medina, I., Casal, J., and Fabre, C.C.G. (2015). Do circadian genes and ambient temperature affect substrate-borne signalling during *Drosophila* courtship? Biol. Open *4*, 1549–1557.

S3. Mamiya, A., Gurung, P., and Tuthill, J.C. (2018). Neural coding of leg proprioception in *Drosophila*. Neuron *100*, 636–650.

S4. Jenett, A., Rubin, G.M., Ngo, T.T.B., Shepherd, D., Murphy, C., Dionne, H., Pfeiffer, B.D., Cavallaro, A., Hall, D., Jeter, J., et al. (2012). A Gal4-driver line resource for *Drosophila* neurobiology. Cell Rep. *2*, 991–1001.

S5. Kim, S.E., Coste, B., Chadha, A., Cook, B., and Patapoutian, A. (2012). The role of *Drosophila* Piezo in mechanical nociception. Nature *483*, 209–212.

S6. Jourjine, N., Mullaney, B.C., Mann, K., and Scott, K. (2016). Coupled sensing of hunger and thirst signals balances sugar and water consumption. Cell *166*, 855–866.

S7. Woo, S.H., Ranade, S., Weyer, A.D., Dubin, A.E., Baba, Y., Qiu, Z., Petrus, M., Miyamoto, T., Reddy, K., Lumpkin, E.A., et al. (2014). Piezo2 is required for Merkel-cell mechanotransduction. Nature *509*, 622–626.

S8. Ramdya, P., Lichocki, P., Cruchet, S., Frisch, L., Tse, W., Floreano, D., and Benton, R. (2015). Mechanosensory interactions drive collective behaviour in *Drosophila*. Nature *450*, 294-298.

S9. Tsubouchi, A., Yano, T., Yokoyama, T.K., Murtin, C., Otsuna, H., and Ito, K. (2017). Topological and modality-specific representation of somatosensory information in the fly brain. Science (80-.). *358*, 615–623.

S10. Mazzoni, V., Anfora, G., and Virant-Doberlet, M. (2013). Substrate vibrations during courtship in three *Drosophila* species. PLoS One *8(11)*.
